# Tap water is one of the drivers that establish and assembly the lactic acid bacterium biota during sourdough preparation

**DOI:** 10.1038/s41598-018-36786-2

**Published:** 2019-01-24

**Authors:** Fabio Minervini, Francesca Rita Dinardo, Maria De Angelis, Marco Gobbetti

**Affiliations:** 1 Department of Soil, Plant and Food Science, University of Bari Aldo Moro, Bari, Italy; 2 Faculty of Science and Technology, Free University of Bozen, Bolzano, Italy

## Abstract

This study aimed at assessing the effect of tap water on the: (i) lactic acid bacteria (LAB) population of a traditional and mature sourdough; and (ii) establishment of LAB community during sourdough preparation. Ten tap water, collected from Italian regions characterized by cultural heritage in leavened baked goods, were used as ingredient for propagating or preparing firm (type I) sourdoughs. The same type and batch of flour, recipe, fermentation temperature and time were used for propagation/preparation, being water the only variable parameter. During nine days of propagation of a traditional and mature Apulian sourdough, LAB cell density did not differ, and the LAB species/strain composition hardly changed, regardless of the water. When the different tap water were used for preparing the corresponding sourdoughs, the values of pH became lower than 4.5 after two to four fermentations. The type of water affected the assembly of the LAB biome. As shown by Principal Components Analysis, LAB population in the sourdoughs and chemical and microbiological features of water used for their preparation partly overlapped. Several correlations were found between sourdough microbiota and water features. These data open the way to future researches about the use of various types of water in bakery industry.

## Introduction

An increasing number of artisan and industrial bakeries make use of sourdough as biological leavening agent and/or baking improver^1^. Sourdough, mainly based on wheat or rye flour and water, is a relatively complex microbial ecosystem densely inhabited by yeasts and lactic acid bacteria (LAB), which are mainly responsible for dough leavening and acidification^2–4^. Despite the simplicity of the operations, the management of the sourdough requires skills, and control during either its preparation or its propagation and use^1^. In particular, the studies of the latter two decades allowed catching the crucial phases that drive the establishment and assembly of sourdough microbial community during both preparation and further propagation and use.

Within this community, LAB have received much more attention than yeasts, because of their higher diversity^5^ and greater influence on odor, aroma, shelf-life and nutritional aspects of sourdough baked goods^6^. Sourdough LAB may originate from flour, additional ingredients and bakery environment. Flour is contaminated by LAB because these bacteria populate the external and internal layers of kernels^5^. Air, insects, operators and milling facilities transfer LAB on the external layers of kernels^7^. LAB populating the internal layers of kernels are thought to be part of cereal endophytic microbiota^8^ and may become dominant in the sourdough ecosystem^9^. House microbiota, i.e. microorganisms contaminating bakery environment, may play the role of additional inoculum for sourdoughs that are daily propagated through back-slopping^6^. In some cases, ingredients of vegetable origin are added in early fermentation steps. The serial fermentations, repeated for 7–15 days, lead to mature sourdough, characterized by stable numbers of LAB and yeasts, and constant acidification and leavening performances^10^. Once mature sourdough has been produced, the stability of its microbial community is debated. Some researchers reported about very stable sourdoughs, at microbial species^11–13^ and even strain level^14,15^. Others observed that bacterial species and strains fluctuate within different time in the same sourdough^16–18^.

So far, no study has been carried out on the eventual effect of water on the establishment and assembly of sourdough microbiota and quality of sourdough bread. Yet, water is the second main ingredient of sourdough and it could be hypothesized that it may somehow affect microbial dynamics^6^. Water allows obtaining the desired viscoelastic properties of gluten and is involved in all the reactions occurring in kneading, fermentation and baking^19^. Hardness (mg of CaCO_3_ in one liter) and pH of water have been regarded as affecting quality of leavened baked goods. Minerals contained in medium hard (100–200 mg/L) water optimally interact with gluten. Overall, they are nutrients required by pro-technological microorganisms^20^. Direct relationships were found between water hardness and (i) bread dough stability time^21^, (ii) specific volume and (iii) softness of steamed bread^22^, whereas inverse correlation was found between water hardness and bread dough degree of softening^21^. Use of water with too high pH (e.g., 8.0) may drive pH of dough far from the optimal values (5.0–6.0) for enzymatic (e.g., amylases) and microbial activities^20^. On the contrary, use of slightly acid water (pH = 5.0–6.0) favors yeast growth and, consequently, causes increased volume of steamed bread^22^. Only anecdotal information, referred by a few artisan bakers, is available about the importance of water on sourdough bread, although some procedural guidelines of typical and/or traditional breads (e.g., Pane di Altamura PDO)^23^ indicate some features of the water to be used in bread-making. Metal ions and trace elements in tap water, mainly originating from “hard water” used in the manufacturing process^24^, could affect the assembly and stability of the sourdough microbiota.

This study aimed to clarify whether tap water affects: (i) LAB population of a traditional and mature sourdough during use; and (ii) establishment of bacterial community during sourdough preparation. For this purpose, ten tap water were used for (i) propagating the same traditional and mature sourdough, and (ii) starting ten new sourdoughs, under the same process parameters (flour, dough yield, percentage of inoculum, time and temperature of fermentation). LAB inhabiting the sourdough were described by culture-dependent and –independent approaches.

## Results

### The chemical and microbiological characteristics markedly differed among tap water from various Italian regions

Tap water were collected (May–June 2017) from the following 10 Italian regions characterized by cultural heritage in leavened baked goods: Abruzzo (coded as A) (e.g., Pane di Cappelli, Pizza di Pasqua), Basilicata (B) (Pane di Matera PGI), Campania (C) (Pane di Montecalvo Irpino, Pane Saragolla), Lazio (LA) (Pane casereccio di Genzano PGI), Lombardia (LO) (Panettone), Puglia (P) (Pane di Altamura PDO, Pane di Laterza), Toscana (T) (Bozza Pratese, Buccellato di Lucca, Pane di Altopascio Tradizionale, Pane Toscano PDO), Trentino-Alto Adige (TA) (Pane di segale), Umbria (U) (Pane di Terni, Torcolo di San Costanzo), and Veneto (V) (Bastone di Padova, Nadalin, Pandoro, Veneziana). Water was collected at public fountains, stored inside clean glass bottles and kept at 4 °C until analyses and use as ingredient for propagating/preparing sourdough.

Fixed residue and electrical conductivity, both directly related with concentration of ions, ranged from ca. 186 (tap water B) to 441 (T) mg/L, and from ca. 291 (B) to 606 (U) μS/cm, respectively (Table 1). Hardness varied from ca. 162 (B) to 343 (LA) mg of CaCO_3_/L. Tap water B and TA were characterized by the lowest (*P* < 0.05) values of electrical conductivity and hardness. Bicarbonate, the most abundant ion in tap water, ranged from 165 (tap water TA) to 400 (LA) mg/L. Calcium was found at the highest (*P* < 0.05) levels in tap water C, T, U and, especially LA. Samples also differed (*P* < 0.05) for the levels of nitrate, chloride, sulphate, sodium, potassium and magnesium. Other ions (bromide, nitrite, phosphate, lithium, sulphites and ammonia) were not detectable. Sodium adsorption ratio (SAR), indicating the sodium content referred to calcium and magnesium, was ever lower than 1, except for tap water A and P. Samples T and U showed the lowest (*P* < 0.05) values of pH (7.3–7.5). On the contrary, tap water A was the most alkaline (pH 8.1). Heterotrophic psychrophilic microorganisms ranged from 1.0 (tap water LO) to 4.2 (tap water B) log CFU/mL, but were not detected in tap water LA, T and V. Heterotrophic mesophilic microorganisms were found at cell density varying from 0.8 (C) to 3.4 (B) log CFU/mL, but were absent in tap water LA, LO and T.

**Table 1:** Chemical and microbiological characteristics of the tap water collected in ten Italian regions^§^.

	A	B	C	LA	LO	P	T	TA	U	V
Fixed residue at 180 °C (mg/L)	292.0 ± 8.1e	186.4 ± 1.5 h	208.8 ± 0.9 g	361.2 ± 9.9bc	380 ± 9.6b	324.0 ± 5.0d	441.2 ± 3.7a	245.2 ± 1.2 f	348.8 ± 9.8c	285.2 ± 7.7e
Electrical conductivity (µS/cm)	453 ± 4.5 g	291 ± 1.0i	545 ± 1.8d	565 ± 5.4c	571 ± 9.9bc	470 ± 5.3 f	585 ± 9.7b	315 ± 2.1 h	606 ± 9.0a	491 ± 8.6e
Bicarbonate (mg/L)	210 ± 9.7gh	195 ± 9.5 h	335 ± 2.7c	400 ± 3.9a	285 ± 9.0e	220 ± 9.6fg	230 ± 9.4 f	165 ± 1.0i	350 ± 8.5b	300 ± 7.1d
Ca^2+^ (mg/L)	56.9 ± 4.0de	53.6 ± 3.8ef	99.8 ± 7.0ab	105.7 ± 7.4a	96 ± 6.7b	62.1 ± 4.3d	101.8 ± 7.1ab	48.3 ± 3.4 f	102.1 ± 7.1ab	70.5 ± 4.9c
NO_4_^2−^ (mg/L)	2.6 ± 0.7c	2 ± 0.5c	1.4 ± 0.4c	2.8 ± 0.7c	34.8 ± 8.7a	3.1 ± 0.8c	2.7 ± 0.7c	5.6 ± 1.4c	30.8 ± 7.7a	18.9 ± 4.7b
Fl^−^ (mg/L)	0.4 ± 0.1a	<0.1c	<0.1c	<0.1c	<0.1c	0.4 ± 0.1a	0.2 ± 0.0a	<0.1c	0.2 ± 0.0a	<0.1c
Cl^−^ (mg/L)	36.3 ± 3.3a	7.6 ± 0.7c	5.8 ± 0.5c	5.7 ± 0.5c	32 ± 2.9a	33.3 ± 3.0a	33.8 ± 3.0a	13.3 ± 1.2c	22.2 ± 2.0b	12 ± 1.1c
SO_4_^2−^ (mg/L)	58.4 ± 7.6b	8.4 ± 1.1 f	3.2 ± 0.4 f	16.5 ± 2.1e	46 ± 6.0c	59.8 ± 7.8b	120 ± 15.6a	35.7 ± 4.6d	34.8 ± 4.5d	25.7 ± 3.3de
Na^+^ (mg/L)	34.3 ± 3.4a	8.3 ± 0.8e	19.1 ± 1.9 cd	4 ± 0.4e	13.6 ± 1.4de	33.7 ± 3.4a	23 ± 2.3bc	10.8 ± 1.1e	30.2 ± 3.0ab	5.9 ± 0.6e
K^+^ (mg/L)	6.7 ± 0.7b	1.7 ± 0.2b	23.8 ± 2.4a	0.9 ± 0.1b	1.6 ± 0.2b	6.7 ± 0.7b	1.7 ± 0.2b	2.8 ± 0.3b	2.5 ± 0.3b	1 ± 0.1b
Mg^2+^ (mg/L)	14.1 ± 1.1bc	6.8 ± 0.5c	4.9 ± 0.4c	19.4 ± 1.6b	20.8 ± 1.7ab	13.9 ± 1.1bc	14.4 ± 1.2bc	11.9 ± 1.0bc	13.4 ± 1.1bc	28.4 ± 2.3a
pH	8.1 ± 0.2a	7.9 ± 0.2b	7.8 ± 0.2bc	7.9 ± 0.2b	7.8 ± 0.2bc	7.7 ± 0.2c	7.5 ± 0.2d	7.8 ± 0.2bc	7.3 ± 0.2e	7.9 ± 0.2b
Hardness (mg of CaCO^3^/L)	200 ± 9.9e	162 ± 8.8 f	269 ± 6.8d	343 ± 6.3a	325 ± 9.0ab	212 ± 9.5e	313 ± 9.7b	169 ± 8.6 f	310 ± 6.7b	293 ± 7.4c
SAR^*^	1.06 ± 0.06a	0.28 ± 0.04e	0.51 ± 0.08c	0.09 ± 0.05 f	0.33 ± 0.06de	1.01 ± 0.05a	0.57 ± 0.09c	0.36 ± 0.05d	0.75 ± 0.0b	0.15 ± 0.07 f
HPC^¥^ at 22 °C	3.3 ± 0.1b	4.2 ± 0.3a	1.6 ± 0.1d	n.d.^†^	1.0 ± 0.2d	2.9 ± 0.2bc	n.d.	3.3 ± 0.3b	2.6 ± 0.3c	n.d.
HPC at 37 °C	2.1 ± 0.2b	3.4 ± 0.2a	0.8c	n.d.	n.d.	2.3 ± 0.3b	n.d.	1.0 ± 0.2c	2.6 ± 0.1b	0.9 ± 0.2c

^§^Each region is coded according to the Results section, first paragraph.

^*^SAR, Sodium adsorption ratio, calculated as the ratio between sodium concentration (expressed in milliequivalent/L) and the square root of half the sum of magnesium and calcium concentrations (expressed in milliequivalent/L).

^¥^HPC, heterotrophic plate count determined at 22 °C (aerobic psychrophilic microorganisms) and 37 °C (aerobic mesophilic microorganisms).

^†^n.d., not detected.

Mean values (±S.D.) in the same row with a common letter were not significantly different at a *P* value of 0.05.

**Figure 1:**
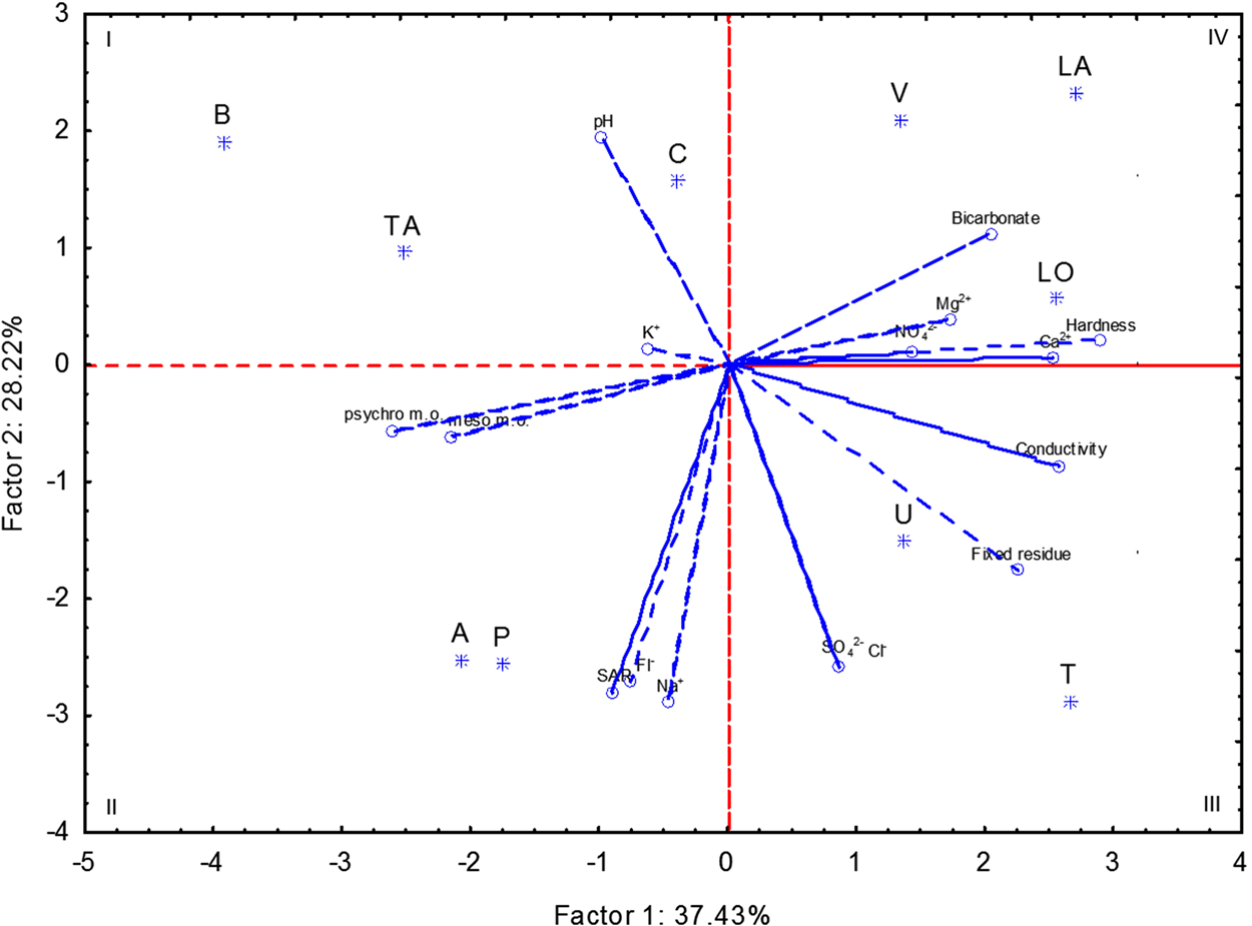
Score and loading plots obtained through Principal Components Analysis performed on the chemical and microbiological characteristics of the water collected in ten Italian regions. SAR, sodium adsorption ratio; psychro m.o., aerobic psychrophilic microorganisms; meso m.o. aerobic mesophilic microorganisms.

Principal Components Analysis (PCA) was carried out using chemical and microbiological characteristics of tap water as data entries. The two first principal components explained ca. 66% of the total variance (Fig. 1). PC1 mainly loaded water bicarbonate, hardness and heterotrophic microbes. PC2 mainly distributed samples according to sodium and sulphate levels. PCA grouped in the first quadrant tap water B, C and TA, being characterized by relatively low fixed residue. Tap water A and P, sharing the highest concentrations of fluoride, sodium and value of SAR, fell in the second quadrant. Tap water U and T were grouped in the third quadrant, because they showed the highest and lowest values of electrical conductivity and pH, respectively. The fourth quadrant grouped tap water LA, LO and V, being characterized by relatively high concentration of magnesium.

### Tap water hardly affected the microbiological features during propagation of a traditional and mature Apulian sourdough

Before propagation, the pH value of the traditional and mature sourdough, produced by P water, was ca. 4.0. Cell density of presumptive LAB and yeasts were ca. 9.0 (both on modified de Man-Rogosa-Sharpe (mMRS) and Sour Dough Bacteria (SDB) agar media) and 7.0 log CFU/g, respectively. The sourdough showed a 400 ml volume increase in 6 h of fermentation (data not shown). It was propagated at laboratory for nine days under the same conditions but using different tap water collected from the other 9 Italian regions. In addition, a control sourdough (P sourdough) was propagated using the P water. After sourdough propagation, the values of pH ranged from 3.96 to 4.03, being not significantly different (*P* > 0.05) from the value before propagation. Similarly, no significant differences (*P* > 0.05) were detectable for the number of LAB, which varied from 8.9 to 9.2 CFU/g. In addition, the values of pH and LAB cell density were not significantly (*P* > 0.05) different among the ten propagated sourdoughs. The yeast cell density decreased (*P* < 0.05) by approximately one log cycle (5.8–6.2 log CFU/g) and, consequently, the leavening capacity decreased (*P* < 0.05) to 300 ml, regardless of the tap water used during sourdough propagation (data not shown).

LAB were isolated from the sourdough before propagation and from the 10 sourdoughs after propagation. As shown by Randomly Amplified Polymorphic DNA (RAPD)-PCR analysis and identification of bacterial isolates, two strains of *Lactobacillus sanfranciscensis* and two strains of *Lactobacillus plantarum* dominated the traditional and mature sourdough before propagation (Supplementary Fig. S1a). Both species were detected in all the sourdoughs after propagation, regardless of the water used. In detail, at least three out of the four lactobacilli strains identified in the sourdough before propagation were also detectable in all the propagated sourdoughs. Besides these strains, the sourdoughs propagated with A or P water harbored another strain of *L*. *plantarum* (s3) and one strain (s1) of *Pediococcus pentosaceus* (Supplementary Fig. S1a,c). *L*. *plantarum* s3 was also found in the sourdoughs propagated with B (Supplementary Fig. S1b) or U water (Supplementary Fig. S1d). The sourdoughs propagated with C, LA (Supplementary Fig. S1b), TA and V (Supplementary Fig. S1d) water, besides harboring the same strains of *L*. *plantarum* and *L*. *sanfranciscensis* detected in the sourdough before propagation, contained *P*. *pentosaceus* s1.

### Tap water affected hardness, gumminess and chewiness of breads made using a traditional and mature sourdough

After nine days of propagation with different water, each traditional and mature sourdough was used as starter (in combination with baker’s yeast) for bread-making. No significant (*P* > 0.05) differences were found among the breads for elasticity (89–100%), cohesiveness (0.29–0.38), specific volume (2.52–2.82 cm^3^/g) and percentage of gas cell area (black pixel area ranging from 54 to 57%) (Table 2). Hardness ranged from ca. 15.5 (bread started with traditional sourdough propagated with tap water P and LO) to 28 N (C), with significant (*P* < 0.05) differences among most of breads. The breads started with sourdough propagated using tap water C, LA and U showed higher (*P* < 0.05) gumminess (43.81 N on average) than the breads obtained with sourdough propagated using tap water LO and P (26.08 N on average). The same three breads were characterized by higher (*P* < 0.05) values of chewiness (average value: 40.33 N) than the breads started with traditional sourdough propagated using tap water A, LO, P and TA (27.39 N).

**Table 2:** Physical characteristics of breads made by traditional and mature sourdough propagated for nine days using tap water collected in ten Italian regions.

Sourdough bread^a^	Hardness (N)	Elasticity (%)	Gumminess (N)	Chewiness (N)	Cohesiveness	Specific volume (cm^3^/g)	Black pixel area^b^ (%)
A	19.06 ± 1.0de	90 ± 10a	32.85 ± 11.01ab	29.71 ± 6.50bc	0.34 ± 0.03a	2.62 ± 0.33a	57 ± 0.8a
B	17.90 ± 0.8e	94 ± 6a	35.55 ± 10.05ab	33.69 ± 9.87abc	0.29 ± 0.05a	2.82 ± 0.23a	55 ± 1.1a
C	28.46 ± 1.4a	90 ± 10a	43.86 ± 9.02a	39.66 ± 8.77a	0.31 ± 0.05a	2.52 ± 0.18a	54 ± 0.9a
LA	25.30 ± 0.9b	92 ± 8a	46.27 ± 8.51a	42.48 ± 8.90a	0.37 ± 0.04a	2.72 ± 0.16a	54 ± 1.4a
LO	16.26 ± 0.6 f	100 ± 0a	26.14 ± 9.44b	26.09 ± 9.69bc	0.32 ± 0.05a	2.74 ± 0.23a	56 ± 2.3a
P	15.48 ± 0.7 f	89 ± 8a	26.03 ± 8.69b	23.27 ± 8.50c	0.34 ± 0.02a	2.60 ± 0.25a	56 ± 5.3a
T	18.06 ± 0.5e	96 ± 3a	34.52 ± 10.22ab	33.11 ± 10.03abc	0.38 ± 0.04a	2.63 ± 0.35a	54 ± 1.4a
TA	18.38 ± 0.6e	91 ± 1a	33.47 ± 9.56ab	30.48 ± 9.45bc	0.36 ± 0.02a	2.73 ± 0.19a	56 ± 2.6a
U	23.78 ± 1.1c	94 ± 5a	41.30 ± 9.88a	38.85 ± 8.66a	0.35 ± 0.05a	2.72 ± 0.18a	55 ± 5.6a
V	20.28 ± 0.8d	94 ± 6a	36.44 ± 9.75ab	34.36 ± 8.98ab	0.36 ± 0.04a	2.74 ± 0.14a	54 ± 1.8a

^a^The name of each sourdough bread derived from the tap water used during the propagation of traditional sourdough.

^b^Black pixel area was the percentage of the image area filled up with black pixel (gas cells).

Mean values (±S.D.) in the same column with a common letter were not significantly different at a *P* value of 0.05.

Correlations between tap water characteristics and bread hardness, gumminess and chewiness were calculated. Positive (*P* < 0.05) correlations (r > 0.70) were found between concentration of bicarbonate in water and the above-mentioned bread textural features. Weaker correlations (r > 0.50) were found between calcium and bread hardness and chewiness, as well as between potassium in water and bread hardness. All the three textural features of bread were negatively correlated (r < −0.63) with chloride in water. In addition, a weaker (r = −0.52) negative correlation was found between sulphate and bread hardness.

### Tap water affected the establishment and assembly of the LAB biome during sourdough preparation

During preparation of the 10 sourdoughs, the values of pH already significantly (*P* < 0.05) differed after the first 8 h of fermentation (Supplementary Fig. S2). Indeed, fermented doughs obtained using tap water T, TA and U showed the lowest values of pH (5.63–5.67), whereas those obtained using tap water A, LA, LO and P showed the highest values (5.92–5.94). The differences of pH among the fermented doughs diminished after the third fermentation (4.43–4.52), but the sourdoughs produced using water T and U were characterized by the lowest values. After the fourth fermentation, all the sourdoughs showed values of pH lower than 4.5. Afterwards, final pH continued to decrease slightly and then reached constant values after the sixth (sourdoughs produced using tap water A, B, C, P, T, U and V) and eighth (LA, LO and TA) fermentation. After the last fermentation, pH values of the sourdoughs ranged from 4.15 to 4.22.

At the beginning of the first fermentation, presumptive LAB ranged from 2.4 to 2.6 (estimated on mMRS) log CFU/g and from 3.4 to 3.6 (estimated on SDB) log CFU/g, with no significant difference (*P* > 0.05) among the doughs (Supplementary Table 1). Presumptive LAB increased of ca. one log cycle after the first fermentation. At the end of the last fermentation (day 9), cell density of LAB estimated on SDB varied from 9.0 (sourdough back-slopped with tap water LO) to 9.6 (tap water TA) log CFU/g. Under our experimental conditions, only two significant differences (*P* < 0.05) were found at the end of the last fermentation: LAB cell density in sourdough LO vs. sourdough TA, estimated on both SDB and mMRS. Within the same mature sourdough, cell density estimated on mMRS did not differ (*P* > 0.05) from that found using SDB. Cell density of yeasts was determined in the sourdoughs and ranged from 5.7 to 6.0 log CFU/g, with no significant difference (*P* > 0.05) (Supplementary Table 1).

After the first fermentation, 75 presumptive LAB isolates were bio-typed by RAPD-PCR analysis and the representative strains were identified by partial sequencing of 16S rRNA and *pheS* genes. All the fermented doughs harbored a LAB consortium composed of *Lactococcus lactis*, *Leuconostoc citreum* and *Weissella confusa* (Supplementary Table 2). In addition, doughs produced by using tap water B, T and TA also harbored *Leuconostoc pseudomesenteroides*. The highest number of strains was found for *W*. *confusa*. Some of them varied depending on the tap water used.

One hundred and twelve Gram-positive, catalase-negative, non-motile, cocci or rods acidifying isolates from the ten mature sourdoughs (after 9 fermentations) were subjected to RAPD-PCR analysis resulting in 31 strains (Supplementary Fig. S3). The following species were identified: *Lactobacillus plantarum* (12 strains), *Pediococcus pentosaceus* (12), *Ln*. *citreum* (5), *Lactobacillus curvatus* (1) and *Pediococcus acidilactici* (1). *L*. *plantarum* was identified in all the sourdoughs and was the only LAB species found in the sourdough prepared using tap water C (Table 3). *P*. *pentosaceus* was found as co-dominant in seven sourdoughs, prepared with tap water B, LA, LO, P, T, TA and U. Besides *L*. *plantarum* and *P*. *pentosaceus*, the sourdoughs obtained using tap water B and LO also harbored *L*. *curvatus*. These three species were flanked by *Ln*. *citreum* in the sourdough obtained using tap water U. *Ln*. *citreum* was also found (together with *L*. *plantarum* and *P*. *pentosaceus*) in the sourdoughs prepared with tap water LA and TA. Besides harboring the previously mentioned four species, the sourdough prepared with tap water T also hosted *P*. *acidilactici*. In the sourdoughs prepared with tap water A and V, three and four, respectively, strains of *L*. *plantarum* were flanked by one strain of *L*. *curvatus*. Within *L*. *plantarum*, some strains were detected in two or more sourdoughs (Table 3). For instance, *L*. *plantarum* s4 and s6 were found in the sourdoughs obtained with tap water B, C and V, s5 was found in the C, LA, LO and V sourdoughs, and s9 and s10 were found in the A and P sourdoughs. Similar results were found for strains of *P*. *pentosaceus*.

**Table 3:** Species and strains of lactic acid bacteria isolated from mature sourdoughs prepared using 10 different tap water, at the end of the last fermentation.

	A^a^	B	C	LA	LO	P	T	TA	U	V
*Lactobacillus curvatus* s1	●	●			●		●		●	●
*Lactobacillus plantarum* s4		●	●							●
*L*. *plantarum* s5			●	●	●					●
*L*. *plantarum* s6		●	●							●
*L*. *plantarum* s7			●							
*L*. *plantarum* s8		●	●							●
*L*. *plantarum* s9	●					●				
*L*. *plantarum* s10	●					●				
*L*. *plantarum* s11	●		●			●				
*L*. *plantarum* s12			●							
*L*. *plantarum* s13								●		
*L*. *plantarum* s14							●		●	
*L*. *plantarum* s15								●		
*Leuconostoc citreum* s1							●			
*Ln*. *citreum* s2							●		●	
*Ln*. *citreum* s3				●						
*Ln*. *citreum* s4								●		
*Ln*. *citreum* s5							●			
*Pediococcus acidilactici* s1							●			
*Pediococcus pentosaceus* s2						●				
*P*. *pentosaceus* s3				●	●					
*P*. *pentosaceus* s4				●	●					
*P*. *pentosaceus* s5		●		●			●	●	●	
*P*. *pentosaceus* s6							●	●	●	
*P*. *pentosaceus* s7								●		
*P*. *pentosaceus* s8								●		
*P*. *pentosaceus* s9							●	●	●	
*P*. *pentosaceus* s10								●		
*P*. *pentosaceus* s11				●	●					
*P*. *pentosaceus* s12				●						
*P*. *pentosaceus* s13				●						

The dot indicates the presence of strains.

^a^The name of each sourdough derived from the tap water used during the preparation of sourdough.

**Figure 2:**
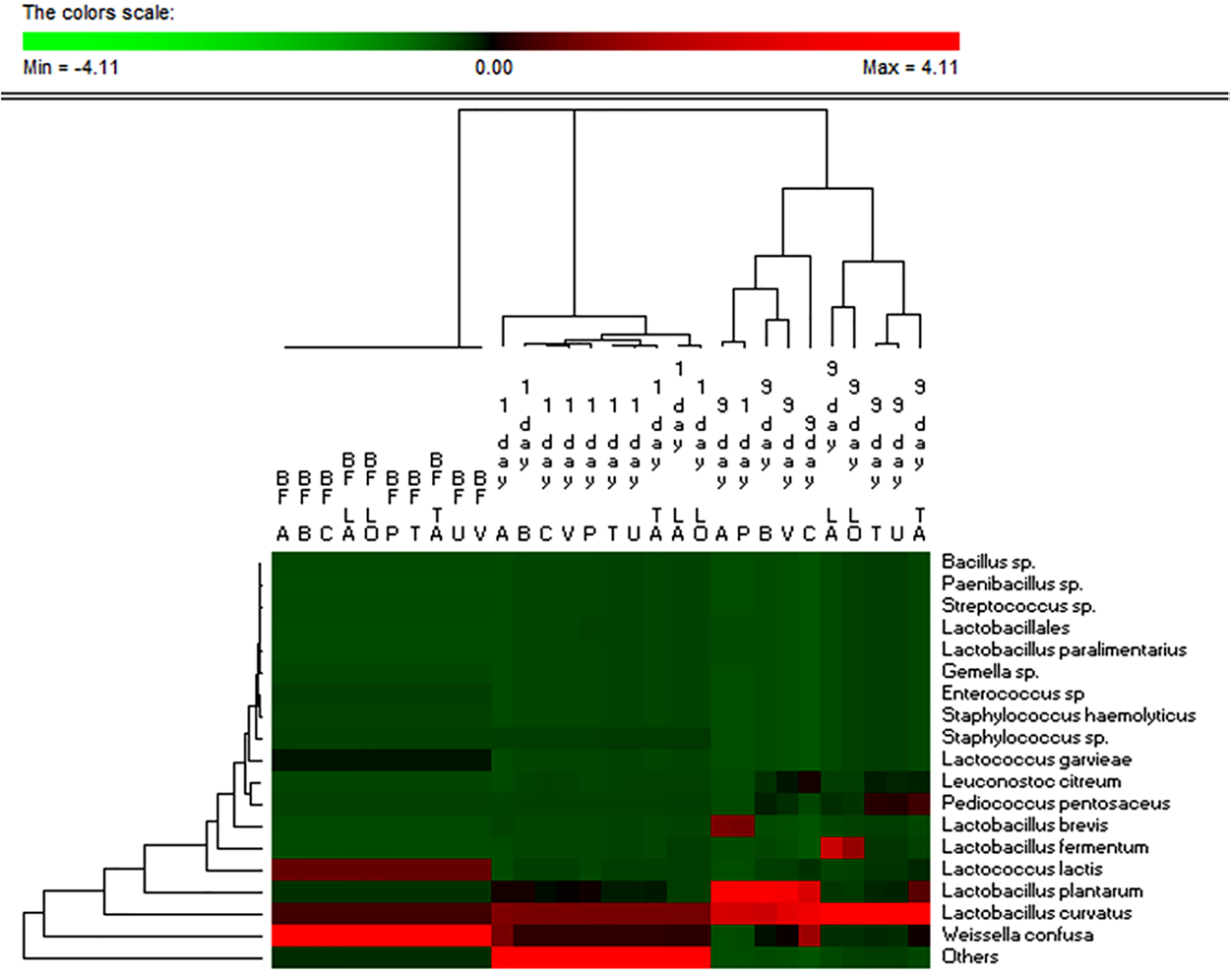
Permutation analysis summarizing the relative abundance of *Firmicutes* species found in dough before fermentation (BF), after 8 h of fermentation (1 day) and in the related sourdoughs (9 day) prepared using water collected in ten Italian regions. Euclidean distance and McQuitty’s criterion were used for clustering. Colors correspond to normalized mean data levels from low (green) to high (red). The color scale, in terms of units of standard deviation, is also shown.

DNA extracted from the doughs was used as template for 16S metagenetic analysis in order to describe the diversity within the *Firmicutes* phylum (Fig. 2). Doughs before fermentation harbored 15 major Operational Taxonomic Units (OTUs), which were ever detected at values of relative abundance that did not differ (*P* > 0.05) among the doughs. *W*. *confusa* was the OTU found at the highest relative abundance (ca. 61%), followed by *Lc*. *lactis* (13.8%), *L*. *curvatus* (9.68%) and *Lactococcus garvieae* (4.41%). After the first fermentation (day 1), *L*. *curvatus*, *W*. *confusa* and *L*. *plantarum* were the dominant OTUs. Compared to doughs after 1 day of fermentation, the relative abundance of *Firmicutes* found in the mature sourdoughs (day 9) changed. Overall, 11 major OTUs were attributed to LAB (Table 4, Fig. 2). Six species (*L*. *curvatus*, *Lactobacillus fermentum*, *L*. *plantarum*, *P*. *pentosaceus*, *Ln*. *citreum* and *W*. *confusa*) were detected in all the sourdoughs, but their relative abundance varied among sourdoughs. When tap water A and P were used for preparing the respective sourdoughs, *L*. *plantarum* was the most abundant OTU (relative abundance of ca. 49%), followed by *L*. *curvatus* (ca. 33%) and *Lactobacillus brevis* (ca. 16%). *L*. *plantarum* and *L*. *curvatus* were also the two most abundant OTUs in the sourdoughs prepared with tap water B, C and V. In these sourdoughs, *W*. *confusa* was also detected at relative abundance ranging from ca. 5 to 19%. *L*. *curvatus* and *L*. *fermentum* were the dominant OTUs in the sourdoughs produced with tap water LA and LO. The sourdoughs produced with tap water T and U were characterized by *L*. *curvatus* at the highest relative abundance (ca. 81%) and by other species (*L*. *plantarum*, *P*. *pentosaceus*, *Ln*. *citreum*, *W*. *confusa* and *Lc*. *lactis*) whose abundance varied between ca. 1 and 8%. When tap water TA was used for preparing sourdough, *L*. *curvatus* was the most abundant OTU (relative abundance of ca. 65%), followed by *L*. *plantarum* (ca. 13%), *P*. *pentosaceus* (ca. 10%) and *W*. *confusa* (ca. 6%).

**Table 4:** Relative abundance (%)^*^ of *Firmicutes* OTU found in the mature sourdoughs prepared using tap water collected in ten Italian regions, at the end of the last fermentation.

	A^a^	B	C	LA	LO	P	T	TA	U	V
*Enterococcus sp*	0.000a	0.004 ± 0.005a	0.000b	0.000b	0.000b	0.000b	0.000b	0.000b	0.000b	0.000b
*Lactobacillus brevis*	16.168 ± 0.718a	0.021 ± 0.002c	0.000c	0.071 ± 0.032c	0.044 ± 0.018c	15.563 ± 0.494a	0.266 ± 0.055b	0.000c	0.233 ± 0.077bc	0.010 ± 0.005c
*Lactobacillus curvatus*	33.921 ± 0.272d	33.632 ± 1.315d	38.790 ± 0.640c	57.783 ± 5.225b	67.709 ± 3.516b	33.528 ± 0.630d	80.679 ± 3.081a	64.886 ± 0.608b	82.100 ± 0.536a	38.548 ± 0.749c
*Lactobacillus fermentum*	0.191 ± 0.040e	0.347 ± 0.003d	0.011 ± 0.025 f	33.675 ± 4.360a	23.006 ± 0.753b	0.131 ± 0.020e	0.865 ± 0.123c	0.541 ± 0.064 cd	0.968 ± 0.134c	0.183 ± 0.033e
*Lactobacillus paralimentarius*	0.322 ± 0.049a	0.000b	0.000b	0.000b	0.000b	0.257 ± 0.149a	0.000b	0.000b	0.000b	0.000b
*Lactobacillus plantarum*	48.512 ± 0.321b	53.595 ± 0.397a	31.798 ± 0.756d	1.825 ± 0.144 g	1.289 ± 0.092 g	49.782 ± 0.807b	2.875 ± 0.206 f	12.620 ± 0.292e	2.882 ± 0.026 f	46.598 ± 0.165c
*Pediococcus pentosaceus*	0.335 ± 0.016 f	3.844 ± 0.120c	1.291 ± 0.249e	2.480 ± 0.147d	1.809 ± 0.352e	0.279 ± 0.044 f	7.992 ± 1.070ab	9.637 ± 0.343a	7.643 ± 0.442b	2.711 ± 0.002d
*Leuconostoc citreum*	0.142 ± 0.009e	2.539 ± 0.605 cd	5.863 ± 0.540a	1.277 ± 0.050d	1.507 ± 0.749d	0.080 ± 0.045 f	3.613 ± 1.085bc	3.471 ± 0.155c	2.847 ± 0.180c	4.532 ± 0.131b
*Weissella confusa*	0.026 ± 0.008d	4.713 ± 0.660b	19.401 ± 0.831a	2.506 ± 0.479c	4.014 ± 2.518bc	0.013 ± 0.019d	2.570 ± 0.168c	5.888 ± 0.001b	2.197 ± 0.054c	5.620 ± 0.469b
*Lactococcus lactis*	0.000e	1.305 ± 0.331bcd	2.845 ± 0.219a	0.384 ± 0.076d	0.524 ± 0.439d	0.000e	1.137 ± 0.370bcd	2.948 ± 0.052a	1.126 ± 0.090 cd	1.799 ± 0.010b
*Lactobacillales*	0.383 ± 0.099a	0.000b	0.000b	0.000b	0.000b	0.366 ± 0.041a	0.003 ± 0.004b	0.000b	0.000b	0.000b
Others	0.000a	0.000a	0.000a	0.000a	0.000a	0.000a	0.000a	0.010 ± 0.003a	0.006 ± 0.002a	0.000a

*Values are the mean (±S.D.) of two analytical replicates performed using DNA extracted from a pool composed of sourdoughs obtained in two parallel and independent series of fermentations. Values in the same row with a common letter were not significantly different at a *P* value of 0.05.

^a^The name of each sourdough derived from the tap water used during the preparation of sourdough.

When the 10 newly prepared sourdoughs were used as starters for bread-making, the resulting breads approximately showed the same differences as those found for breads obtained with traditional sourdough propagated using different tap water (Supplementary Table 3).

### Correlations between sourdough microbiota and chemical composition of tap water

**Figure 3:**
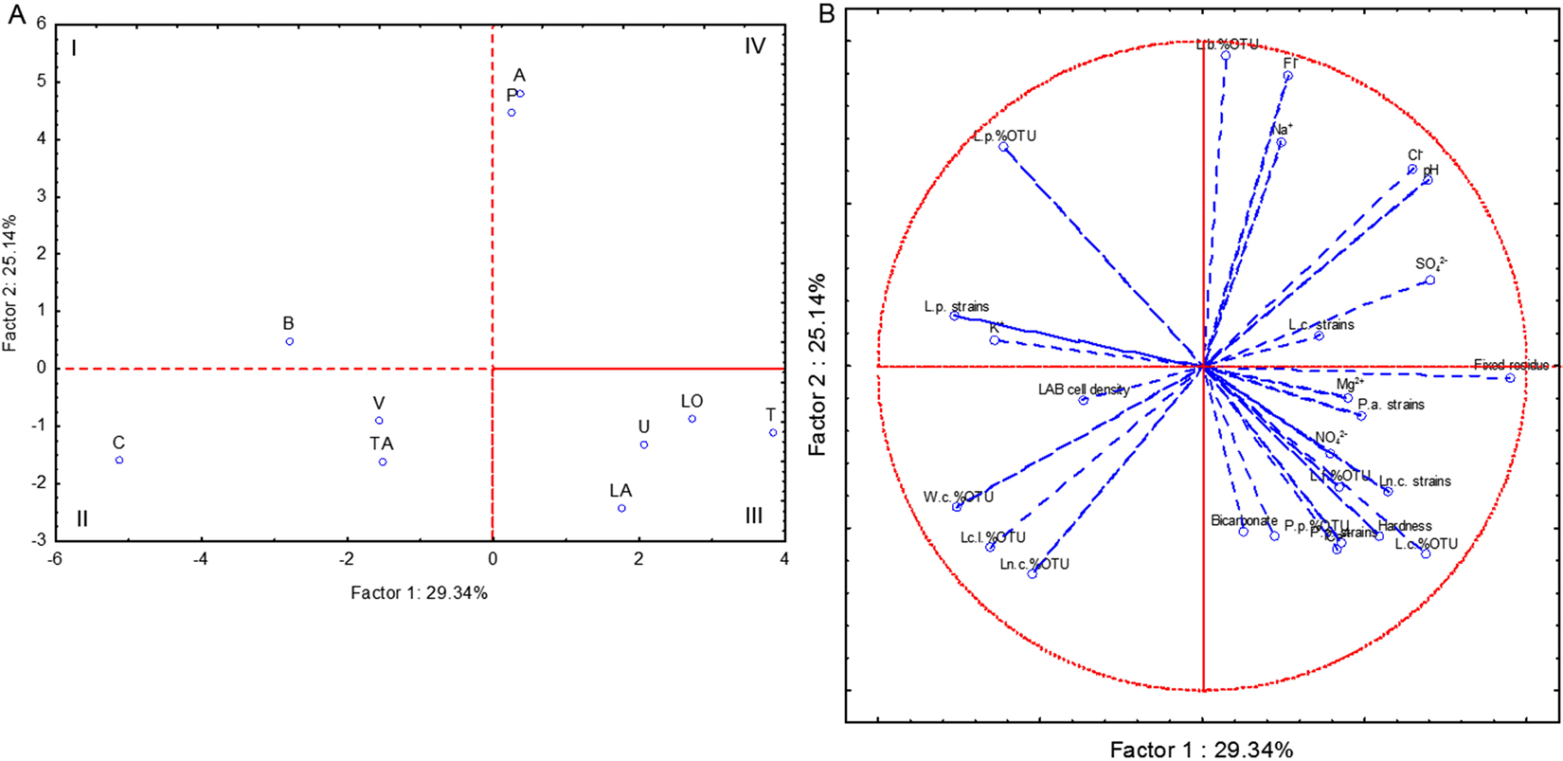
Score (**A**) and loading (**B**) plots of first and second principal components after Principal Component Analysis performed on the chemical characteristics of the water collected in ten Italian regions and on the microbiological characteristics of the sourdoughs prepared at laboratory. L.p. strains, number of strains of *Lactobacillus plantarum*; L.c. strains, number of *Lactobacillus curvatus* strains; P.p. strains, number of *Pediococcus pentosaceus* strains; P.a. strains, number of *Pediococcus acidilactici* strains; Ln. c. strains, number of *Leuconostoc citreum* strains; W.c.%OTU, relative abundance of *Weissella confusa*; L.p.%OTU, relative abundance of *Lactobacillus plantarum*; L.f.%OTU, relative abundance of *Lactobacillus fermentum*; L.c.%OTU, relative abundance of *Lactobacillus curvatus*; P.p%OTU, relative abundance of *Pediococcus pentosaceus*; Ln.c.%OTU, relative abundance of *Leuconostoc citreum*; Lc.l.%OTU, relative abundance of *Lactococcus lactis*.

PCA based on the chemical characteristics of water and on the microbiological characteristics (LAB cell density, percentage of strains per species, relative abundance of OTU) of the mature sourdoughs showed that two first principal components explained ca. 54% of the total variance (Fig. 3). Sourdoughs produced with tap water C, V and TA were grouped in the second quadrant because of the highest values of relative abundance of *W*. *confusa*, *Lc*. *lactis* and *Ln*. *citreum*. Sourdoughs prepared with tap water LA, LO, T and U fell in the third quadrant. They were characterized by the highest relative abundance of *L*. *curvatus* and *L*. *fermentum* and presence of strains allotted to *P*. *pentosaceus*. Sourdoughs A and P, characterized by the highest relative abundance of *L*. *brevis*, fell in the fourth quadrant. Positive correlations (*P* < 0.05) were found between concentration of potassium in tap water and number of *L*. *plantarum* strains (r = 0.86) and relative abundance of *W*. *confusa* (r = 0.80). On the contrary, potassium and *L*. *curvatus* were negatively correlated (r = −0.63). Magnesium concentration in tap water was correlated positively with *L*. *fermentum* (r = 0.69). Relative abundances of *Ln*. *citreum*, *W*. *confusa* and *Lc*. *lactis* were negatively correlated with magnesium and, especially, chloride (r ≤ −0.60). Positive correlations (*P* < 0.05) were found between sodium and relative abundance of *L*. *brevis* (r = 0.78) and *L*. *plantarum* (r = 0.25). Sodium was negatively correlated with *L*. *fermentum* (r = −0.69). Concentration of sulphate in water was positively correlated with number of *P*. *pentosaceus* (r = 0.82) and *Ln*. *citreum* (r = 0.69) strains. Bicarbonate and relative abundance of *L*. *fermentum* were also correlated (r = 0.75).

## Discussion

In this study ten tap water, collected from as many Italian regions characterized by cultural heritage in leavened baked goods, were used for propagating or preparing firm (type I) sourdoughs. All the tap water used in this study could be classified as either hard (120–300 mg of CaCO_3_/L; water A, B, C, P, TA, V) or very hard (more than 300 mg/L; the remaining ones). Although some researchers claimed that hardness of water should range from 50 to 200 mg/L^25^, in Italy, one of the major countries in the world in terms of bread cultural heritage, water evidently show, on average, hardness values above that range. When trying to assess whether tap water would influence sourdough, we found two fairly opposed effects, depending on the sourdough protocol of use (propagation vs. preparation). When different tap water were used during nine days of laboratory propagation of a traditional and mature Apulian sourdough, LAB cell density did not differ, regardless of the type of tap water used. Regarding the composition of LAB population of the propagated sourdoughs, around 60–80% of all the strains were identical to those detected before propagation. Previously, it had been shown that five out of seven traditional sourdoughs, propagated for 20 days under laboratory conditions, harbored from 50 to 100% of the LAB strains detected before starting the propagation^18^. However, in the current study two strains belonging to *L*. *plantarum* and *P*. *pentosaceus*, which had not been detected in the sourdough before propagation, were found in eight propagated sourdoughs. The retrieval of these additional LAB could be explained by temporary fluctuations, although we cannot exclude that these LAB could have the chance to replace the lactobacilli strains that dominate the traditional sourdough^6^. The results found in the current study showed that the type of tap water used during propagation of a mature and stable artisan sourdough had modest effect on the LAB population and, consequently, on the acidifying capacity. During laboratory propagation, cell density of yeasts decreased. This could be due to the place of sourdough propagation, being laboratories less subjected than bakeries to environmental contamination with baker’s yeast^18^.

In order to scout the effect of tap water on sourdough bread, the propagated sourdoughs were used for making breads, in combination with a low concentration of baker’s yeast. The breads differed for some textural features. Overall, bread texture results from interactions between gluten matrix (whose behavior differs depending on flour), type of leavening agent used, fermentation conditions and chemical composition of tap water^26^. In the experimental design of this study, the tap water used for sourdough propagation, preparation and bread-making was the only variable. Thus, the differences among breads in terms of gumminess, chewiness and hardness might be attributed to tap water. These textural attributes positively correlated with the concentration of bicarbonate, but negatively with chloride. Other weaker positive correlations were between bread hardness and chewiness, and concentration of calcium. Besides, bread hardness also positively correlated with the concentration of potassium. Previous studies showed that pH and hardness of tap water affect both rheological behavior of dough^21^ and texture of bread^20,22^. Water hardness indicates its content of calcium and magnesium^26^. In detail, temporary hardness refers to calcium and magnesium bicarbonates, which decrease upon boiling, whereas permanent hardness, not affected by boiling, refers to calcium and magnesium sulphates^26^. In the current study, relatively weak positive correlations were found between water hardness and bread textural features. This could probably be due to the fact that values of hardness determined for the ten tap water express calcium, magnesium, bicarbonate and sulphate (total hardness), but magnesium in water varied at much lesser extent than calcium and bicarbonate.

When tap water were used for preparing the corresponding sourdoughs, the values of pH became lower than 4.5 after two to four fermentations (back-slopping), depending on the tap water. These values of pH limit the growth of many microorganisms that normally contaminate cereal-based doughs, such as some members of the *Enterobacteriaceae* family. Given that the same batch of flour was used, the reasons for different pH values found during early fermentations should be searched among: (i) different pH and buffering capacity of water; and (ii) specific effect of minerals on the fermentative metabolism of LAB^27^. Tap water T and U had the lowest pH and relatively high total concentration of cations, which overall favor acidification. Regarding the effect of minerals on LAB, no clear relationships were found at the early fermentation steps. However, some minerals (e.g., sulphate, sodium, potassium and magnesium) are normally contained in the media routinely used for LAB cultivation^28^. Although the concentration of most of minerals (especially potassium and magnesium) in flour is much higher than in tap water^29^, some of them could not be available to microorganisms, because sequestered by flour components. For instance, phytic acid in cereals chelates magnesium and other divalent cations^26^. It may be hypothesized that tap water used in dough preparation, along with flour, could provide LAB with minerals necessary to their growth. Yet, such a hypothesis would fall before the results obtained when different tap water were used during propagation of a traditional sourdough. In order to solve this issue, we hypothesize that minerals provided by water (besides those by flour) could drive LAB population just during preparation of sourdough, whose early fermentations are due to bacteria present at low cell density. *Vice versa*, the influence of minerals from tap water would be negligible during propagation of a mature sourdough, inhabited by high microbial numbers.

Based on culture-dependent analysis, the newly prepared sourdoughs harbored from one to five species of LAB. *L*. *plantarum* and *P*. *pentosaceus* were the most commonly isolated species, whereas *L*. *curvatus*, *Ln*. *citreum* and *P*. *acidilactici* were occasionally isolated. *L*. *plantarum* and *P*. *pentosaceus* have been detected in 43% and 14%, respectively, of more than 500 sourdoughs^5^. Upon culture-independent analysis, the sourdoughs were distinguished based on relative abundance of eight LAB OTUs, among which *L*. *plantarum* and *L*. *curvatus* were the most important. Compared to the results from culture-independent analysis, *L*. *curvatus* was isolated at lower frequency, whereas *L*. *plantarum* was more frequently isolated. We hypothesize that *L*. *curvatus* would grow in SDB medium at lower extent than *L*. *plantarum*. Indeed, the medium chosen for culture-dependent analysis may affect the results describing sourdough bacterial diversity^30^. As shown by PCA, LAB population in the sourdoughs and chemical and microbiological features of tap water used for their preparation partly overlapped. This occurred for tap water A and P and the corresponding sourdoughs. Water LA, LO, T and U, sharing high values of hardness and electrical conductivity (PCA in Fig. 1), gave sourdoughs which harbored *L*. *curvatus* at high relative abundance and strains of *P*. *pentosaceus*. In some cases, correlations found between relative abundance of LAB OTUs and chemical features of tap water would suggest that some minerals, such as magnesium, potassium, sodium and sulphate, drive the establishment and assembly of the sourdough LAB biota. Microbial cells need magnesium for several biochemical processes (e.g., biosynthesis of nucleic acids and proteins). Bacteria may show different ability to accumulate magnesium in the cytoplasm. It has been reported that *L*. *brevis* accumulates higher concentration of this mineral than *L*. *plantarum*^31^. This different ability was reflected in higher magnesium concentration in bread started with *Saccharomyces cerevisiae* and *L*. *brevis*^32^. Compared to sodium chloride, magnesium sulphate caused faster growth of *Lactobacillus sakei* in MRS^33^. Although milk contains sufficient magnesium for microbial growth, an exogenous surplus (ca. 16–50% of the normal supply in milk) of this mineral stimulated growth of two streptococci^34^. Potassium is another macro-nutrient for microbial cells, because it is involved in many enzyme activities^27^. In this study, we found that potassium was positively correlated with one member of the *Leuconostocaceae* family. Potassium (along with manganese and phosphate) is essential for growth of *Leuconostoc mesenteroides*. Cell density of LAB was higher in cheeses wherein potassium chloride partially replaced sodium chloride^35^. Similarly, potassium chloride showed lower inhibitory effect than sodium chloride against *Lactobacillus casei*^36,37^. Conversely, it has been recently reported that cheeses wherein sodium was partially replaced with potassium contained lower cell number of *Lc*. *lactis* and *L*. *casei*^38^. Overall, these studies suggest that the same mineral may have different effects depending on the bacterial species.

This study showed that the type of tap water drives the assembly of the LAB biota in newly prepared sourdoughs, but hardly influences the microbiota of stable sourdough. Although we found different counts of heterotrophic microorganisms in the tap water used in this study, the role of water microbiota during sourdough preparation could perhaps be neglected, because of lower level of microbial contamination, compared to that of doughs during fermentation. On the contrary, we found that water mineral concentrations could be an additional driver, along with flour and fermentation parameters, for sourdough bacterial biota establishment.

## Methods

### Chemical and microbiological analyses on tap water

The following chemical analyses were performed on 500 ml of water by SAMER laboratory (Servizio analisi chimiche-merceologiche, Bari): fixed residue at 180 °C^39^; electrical conductivity^40^; bicarbonate (determination by titration, according to an internal method developed by CNR-IRSA); calcium, lithium, sodium, ammonium, potassium, magnesium, nitrate, fluoride, chloride, bromide, nitrite, phosphate and sulphate (through ionic chromatography)^41^; sulphites^42^; pH^43^; hardness^44^. In addition, the SAR was calculated using the following formula:

1 $$SAR=sodium/\sqrt{\frac{1}{2}}(calcium+magnesium).$$

Microbiological analyses were performed by pour-plating water samples on yeast agar (yeast extract 3 g/L, tryptone 6 g/L, agar 15 g/L, pH 7.2 ± 0.2). Colonies were counted after incubation at 22 °C (psychrophilic) and 37 °C (mesophilic) for 72 and 48 h, respectively, thus obtaining the cell density of heterotrophic microorganisms^45^.

### Propagation of a traditional and mature sourdough with different tap water

An Apulian, durum wheat semolina 20-years-old, type I sourdough (dough yield, DY, of 160), dominated by *Lactobacillus sanfranciscensis* s1, *L*. *sanfranciscensis* s2, *Lactobacillus plantarum* s1, *L*. *plantarum* s2 and *Saccharomyces cerevisiae* s1, was continuously propagated for nine days, using the same batch of durum wheat semolina (Grandi Molini Italiani, brand “Ca’ Bianca”, Rovigo, Italy) but ten different tap water. One of these water, namely that collected in Puglia (P), was the same used for conventional propagation of the Apulian traditional and mature sourdough considered in this study. The gross composition of semolina (*alias* “flour”) was the following: moisture, 12.9%; protein, 11.1%; carbohydrates, 71.0%; fiber, 3.5%, fat, 1.5%. Propagation was performed for nine days because we reasonably expected to find the (eventual) effect of water on sourdough in such a lapse of time. The following parameters were adopted in the propagation (back-slopping): DY, 160; inoculum ratio, 20%, fermentation temperature, 30 °C; fermentation time, 6 h; resting time (at 4 °C), 18 h. Before starting propagation and after the last fermentation (day 9), pH, volume increase (in ml), cell densities of LAB and yeasts were monitored in the sourdoughs.

### Sourdough preparation

**Figure 4:**
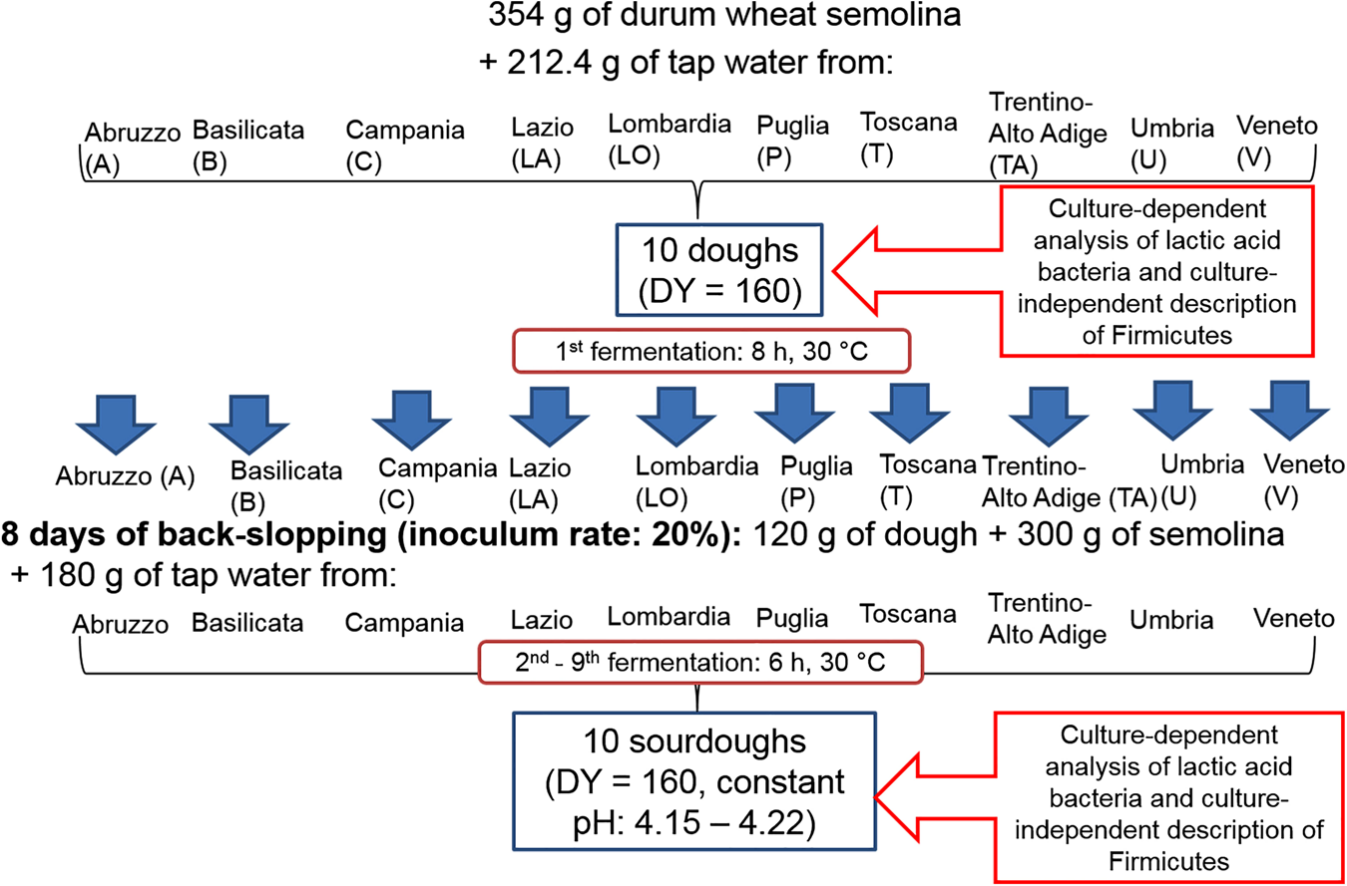
Schematic design showing the production of sourdoughs at laboratory and the analyses performed.

The ten tap water were used for preparing as many sourdoughs, according to the scheme in Fig. 4. Before fermentation, 10 g of dough were sampled and stored at −20 °C for extraction of total DNA. After fermentation (30 °C, 8 h), 10 g of dough were sampled, whereas the remaining dough was stored at 4 °C for 16 h (resting). Upon the 1^st^ back-slopping (inoculum ratio, 20%; DY, 160), dough was fermented (30 °C, 6 h) and then stored at 4 °C for 18 h. After this second fermentation, other seven fermentation steps followed, keeping the same inoculum ratio, DY and conditions of fermentation. At the end of the last (ninth) fermentation, 10 g of dough were sampled. Based on the available literature, a number of back-slopping steps ranging from 5 to 10 is usually needed to obtain mature sourdough^5,46^. Serial fermentations were performed in duplicate. pH was measured at the beginning and end of each fermentation. Presumptive LAB were enumerated in all the doughs at the beginning and end of the first fermentation and at the end of the ninth fermentation. Yeasts were enumerated at the end of the ninth fermentation.

### Acidification and microbial cell densities in doughs and sourdoughs

The values of pH were determined by direct insertion of a Foodtrode (Hamilton, Bonaduz, Switzerland) electrode. Before plate count, the two biological replicates for type of dough produced by using different water were pooled. Each pool was homogenized, serially diluted with sterile peptone water and plate counted. LAB were enumerated using modified de Man-Rogosa-Sharpe (mMRS) (Oxoid, Basingstoke, Hampshire, UK) and Sour Dough Bacteria (SDB) agar media, both supplemented with cycloheximide (0.1 g/L). Yeasts were enumerated using Sabouraud Dextrose agar (SDA) supplemented with chloramphenicol (0.1 g/L)^47^.

### Isolation, identification and biotyping of LAB in sourdoughs

Ten colonies from SDB agar plates inoculated with the intermediate dilution of each single dough (obtained on the first day of sourdough production) and sourdough (traditional sourdough before and after propagation and sourdoughs prepared in laboratory) were randomly picked up to isolate LAB. Isolation of pure bacterial cultures was obtained upon two repeated streaks, using SDB broth. DNA was extracted from Gram-positive, catalase-negative cocci and rods able to acidify the medium, using the Qiagen (Courtaboeuf, France) DNeasy Blood and Tissue Kit, following the manufacturer’s instructions^48^. LAB were biotyped through RAPD-PCR using P4, P7 and M13 primers, applying the same conditions previously described^17,49^. The PCR products (3 µl) were separated by electrophoresis at 100 V for 120 min on 1% (w/v) agarose gel containing (0.005%, v/v) GelRed® Nucleic Acid Gel Stain (Biotium, Inc., Fremont, CA), for detecting the DNA by UV trans-illumination. The molecular sizes of the amplified DNA fragments were estimated by comparison with the HyperLadder^TM^ 1 kb (Bioline Reagents Limited, London, UK). RAPD-PCR profiles were acquired by Gel Doc EQ System (Bio-Rad, Hercules, CA) and compared using Fingerprinting II Informatix^TM^ Software (Bio-Rad). Representative LAB strains were identified using LpigF and LpigR primers targeting 16S rDNA gene^50^ and, when necessary, *recA*^51^, *pheS*^52^ and three couples of primers targeting the 16S/23S spacer region, in order to discriminate species within the *Lactobacillus sakei* group^53^.

### Extraction of total DNA from sourdoughs and Illumina MiSeq analysis

10 g of doughs and related sourdoughs were homogenized with 90 ml of sterile saline solution. Pellet was treated with FastPrep and DNA was extracted using the FastDNA Spin Kit for Soil (MP Biomedicals, Illkrich, France), following the manufacturer’s instructions^54^. Concentration and purity of DNA was assessed spectrophotometrically using the Nanodrop ND-1000 (Thermo Fisher Scientific Inc.). DNA was used as template in PCR using primers Firm350F/Firm814R, targeting the region V3-V4 of *Firmicutes*^55^. PCR were run by RTL Genomics (Lubbock, TX), using internal protocols. PCR products were sequenced at RTL Genomics, using the Illumina 2 × 300 bp paired-end MiSeq platform. Processing of sequenced reads was carried out using, in sequence, the PEAR Illumina paired-end read merger^56^, UCHIME software^57^, USEARCH global alignment algorithm^58^, and UPARSE OTU selection algorithm^59^, as previously described^8^. Operational Taxonomic Units (OTUs) were identified using a database of high quality sequences derived from the NCBI.

### Bread-making

Both traditional and mature, and the newly prepared sourdoughs were used as starters in bread-making. In this latter case, the sourdoughs were considered to be mature after nine fermentations. In detail, 60 g of sourdough (10% inoculum rate) was used as ingredient in bread dough together with 337.5 g of durum wheat semolina (the same type and batch used during sourdough propagation/preparation), 202.5 g of tap water (differing depending on the sourdough used) and 1.5 g (corresponding to 0.25% inoculum rate) of baker’s yeast (Zeus IBA s.r.l. Firenze, Italy). Two loaves per sourdough bread were shaped, each weighing ca. 250 g. After fermentation (30 °C, 3 h) and baking (220 °C, 25 min), bread loaves were cooled at room temperature for 18 h.

### Analyses of breads

Specific volume (cm^3^/g) was determined using the rapeseed displacement method^60^. Breads were subjected to Texture Profile Analysis (TPA) by using the Universal Testing Machine 3344 (Instron, Norwood, USA), equipped with a cylindrical (diameter, 3.6 cm) probe and a 1 kN load cell. Bread loaves were compressed (30%, two cycles) and the following parameters were registered: hardness, defined as the peak force (expressed in N) during the first compression cycle; elasticity, defined as the elastic recovery (expressed as percentage) that occurs when the compressive force is removed; gumminess (expressed in N); chewiness (expressed in N); cohesiveness, defined as the non-dimensional ratio of the positive force area during the second compression cycle to the positive force area recorded during the first compression cycle.

Bread loaves not used for TPA were sliced and the relative images were scanned full-scale using an Image Scanner (Amersham Pharmacia Biotech, Uppsala, Sweden), at 300 dots per inch, and analyzed in grey scale (0–255). Image analysis was performed using the UTHSCSA ImageTool program (Version 3.0, University of Texas Health Science Centre, San Antonio, Texas, freely downloadable at, http://en.bio-soft.net/draw/ImageTool.html)^61^.

### Statistical analyses

Two parallel and independent series of fermentations were run for type of water. One-way ANOVA was applied to the data from two biological replicates (analyzed in triplicate for getting the results about pH and volume increase of sourdough, and physical features of bread) and Tukey’s procedure (at *P* = 0.05) was used to perform pair comparison of treatment means. In addition, the following data were subjected to PCA, using Statistica 7.0 for Windows, and cluster analysis, using PermutMatrix^62^: (i) chemical and microbiological characteristics of the water; (ii) characteristics of sourdough bacterial community (assessed through culture-dependent and – independent methods). Spearman correlations between chemical characteristics of the water and OTUs found in the newly prepared sourdoughs were computed using Statistica v. 7.0.

## Electronic supplementary material

Supplementary figures and tables

## Data Availability

The raw data supporting the conclusions of this manuscript will be made available by the authors, without undue reservation, to any qualified researcher.

## References

[1] Gänzle MG, Ripari V (2016). Composition and function of sourdough microbiota: from ecological theory to bread quality. Int. J. Food Microbiol..

[2] De Vuyst L, Neysens P (2005). Biodiversity of sourdough lactic acid bacteria. Trends Food Sci. Technol..

[3] Gobbetti M (1998). Interactions between lactic acid bacteria and yeasts in sourdoughs. Trends Food Sci. Technol..

[4] Vogel RF (1999). Non-dairy lactic fermentations: the cereal world. Antonie van Leeuwenhoek.

[5] Van Kerrebroeck S, Maes D, De Vuyst L (2017). Sourdoughs as a function of their species diversity and process conditions, a meta-analysis. Trends Food Sci. Technol..

[6] Gobbetti M, Minervini F, Pontonio E, Di Cagno R, De Angelis M (2016). Drivers for the establishment and composition of the sourdough lactic acid bacteria biota. Int. J. Food Microbiol..

[7] Sabillón L, Bianchini A (2016). From field to table: a review on the microbiological quality and safety of wheat-based products. Cereal Chem..

[8] Minervini F (2015). Lactic acid bacteria in durum wheat flour are endophytic components of the plant during its entire life cycle. Appl. Environ. Microbiol..

[9] Minervini F, Lattanzi A, Dinardo FR, De Angelis M, Gobbetti M (2018). Wheat endophytic lactobacilli drive the microbial and biochemical features of sourdoughs. Food Microbiol..

[10] Ripari V, Gänzle MG, Berardi E (2016). Evolution of sourdough microbiota in spontaneous sourdoughs started with different plant materials. Int. J. Food Microbiol..

[11] Scheirlinck I (2008). Taxonomic structure and stability of the bacterial community in Belgian sourdough ecosystems as assessed by culture and population fingerprinting. Appl. Environ. Microbiol..

[12] Vera A, Ly-Chatain MH, Rigobello V, Demarigny Y (2012). Description of a French natural wheat sourdough over 10 consecutive days focusing on the lactobacilli present in the microbiota. Antonie van Leeuwenhoek.

[13] Viiard E, Mihhalevski A, Rühka T, Paalme T, Sarand I (2012). Evaluation of the microbial community in industrial rye sourdough upon continuous back-slopping propagation revealed *Lactobacillus helveticus* as the dominant species. J. Appl. Microbiol..

[14] Gänzle MG, Vogel RF (2003). Contribution of reutericyclin production to the stable persistence of *Lactobacillus reuteri* in an industrial sourdough fermentation. Int. J. Food Microbiol..

[15] Vogel RF (2011). Genomic analysis reveals *Lactobacillus sanfranciscensis* as stable element in traditional sourdoughs. Microb. Cell Fact..

[16] Viiard E (2016). Diversity and stability of lactic acid bacteria in rye sourdoughs of four bakeries with different propagation parameters. Plos One.

[17] Siragusa S (2009). Taxonomic structure and monitoring of the dominant population of lactic acid bacteria during wheat flour sourdough type I propagation using *Lactobacillus sanfranciscensis* starters. Appl. Environ. Microbiol..

[18] Minervini F, Lattanzi A, De Angelis M, Di Cagno R, Gobbetti M (2012). Influence of artisan bakery- or laboratory-propagated sourdoughs on the diversity of lactic acid bacterium and yeast microbiotas. Appl. Environ. Microbiol..

[19] Guzmán C, Posadas-Romano G, Hernández-Espinosa N, Morales-Dorantes A, Peña RJ (2015). A new standard water absorption criteria based on solvent retention capacity (SRC) to determine dough mixing properties, viscoelasticity, and bread-making quality. J. Cereal Sci..

[20] Sinani V, Sana M, Seferi E, Sinani A (2014). The impact of natural water quality on baking products in albania. J. Water Resource Prot..

[21] Ștefan EM, Voicu G, Constantin GA, Ferdeş M, Gheorghe M (2015). The effect of water hardness on rheological behaviour of dough. J. Engin. Studies Res..

[22] Yuru B, Xianlun W (2011). Research on water’s influences on the quality of frozen dough. Procedia Environ. Sci..

[23] European Union (2003). EC Regulation 1291/2003, (July 19). Official J of Europ Union L 181.

[24] Stratford, M. & James, S. A. Non-alcoholic beverages and yeasts in Boekhout, T. & Robert, V. (eds), *Yeasts in Food*. B. Behr’s Verlag GmbH & Co, Hamburg, Germany p. 309–345 (2003).

[25] Banu, C. Tratat de industrie alimentară – Tehnologii alimentare. *ASAB*, Bucarest (2009).

[26] Serna-Saldivar, S. O. Cereal Grains: properties, processing, and nutritional attributes. *CRC Press, Taylor & Francis Group*, LLC, Boca Raton, FL (2010).

[27] Boyaval P (1989). Lactic acid bacteria and metal ions. Dairy Sci. Technol..

[28] Watanabe M, van der Veen S, Abee T (2012). Impact of respiration on resistance of *Lactobacillus plantarum* WCFS1 to acid stress. Appl. Environm. Microbiol..

[29] Ragaee S, El-Sayed MAA, Maher N (2006). Antioxidant activity and nutrient composition of selected cereals for food use. Food Chem..

[30] Vera A, Rigobello V, Demarigny Y (2009). Comparative study of culture media used for sourdough lactobacilli. Food Microbiol..

[31] Roman, J., Gniewosz, M. & Mantorska, J. Porównanie wiązania magnezu, wzrostu i właściwości kwaszących *Lactobacillus brevis* i *Lactobacillus plantarum* w środowisku o podwyższonym stężeniu magnezu. *Acta Scientiarum Polonorum Biotechnologia* **8**, 27–36 (in Polish) (2009).

[32] Gniewosz, M., Chlebowska-Smigiel, A., Lipinska, E., Krasniewska, K. & Rapacka, M. Wpływ zwiększonej zawartości magnezu w hodowlach wybranych szczepów bakterii i drożdży na wybrane cechy jakościowe ciasta i pieczywa. *Zeszyty Problemowe Poste˛pów Nauk olniczych* **578**, 39–47 (in Polish) (2014).

[33] Samapundo S (2010). Influence of NaCl reduction and replacement on the growth of *Lactobacillus sakei* in broth, cooked ham and white sauce. Int. J. Food Microbiol..

[34] Amouzou KS, Prevost H, Divies C (1985). Influence de la supplémentation du lait en magnésium sur la fermentation lactique réalisée par *S*. *lactis* et *S*. *thermophilus*. Dairy Sci. Technol..

[35] Ayyash MM, Sherkat F, Shah NP (2012). The effect of NaCl substitution with KCl on Akawi cheese: chemical composition, proteolysis, angiotensin-converting enzyme inhibitory activity, probiotic survival, texture profile, and sensory properties. J. Dairy Sci..

[36] Cruz AG (2011). Cheeses with reduced sodium content: effects on functionality, public health benefits and sensory properties. Trends Food Sci. Technol..

[37] Karimi R, Mortazavian AM, Karami M (2012). Incorporation of *Lactobacillus casei* in Iranian ultrafiltered Feta cheese made by partial replacement of NaCl with KCl. J. Dairy Sci..

[38] Silva HLA (2018). Partial substitution of NaCl by KCl and addition of flavor enhancers on probiotic Prato cheese: a study covering manufacturing, ripening and storage time. Food Chem..

[39] Istituto Superiore di Sanità. Reference analytical methods for water intended for human consumption according to the Italian Legislative Decree 31/2001, **7**, 65–67. In *Ottaviani M*, *Bonadonna L* (*ed*.), *Chemical methods*, *Rapporti ISTISAN07/31* (2007).

[40] Agenzia per la protezione dell’ambiente e per i servizi tecnici, Consiglio Nazionale delle Ricerche - Istituto di Ricerca sulle Acque. Metodi analitici per le acque. **1**, 131–135. *I*.*G*.*E*.*R*. Roma, Italy (2003).

[41] Agenzia per la protezione dell’ambiente e per i servizi tecnici, Consiglio Nazionale delle Ricerche - Istituto di Ricerca sulle Acque. Metodi analitici per le acque. **1**, 215–224. *I*.*G*.*E*.*R*. Roma, Italy (2003).

[42] American Public Health Association. Standard Methods for Examination of Water & Wastewater. 21st ed. Washington, DC (2005).

[43] Agenzia per la protezione dell’ambiente e per i servizi tecnici, Consiglio Nazionale delle Ricerche - Istituto di Ricerca sulle Acque. Metodi analitici per le acque. **1**, 145–152. *I*.*G*.*E*.*R*, Roma (2003).

[44] Agenzia per la protezione dell’ambiente e per i servizi tecnici, Consiglio Nazionale delle Ricerche - Istituto di Ricerca sulle Acque. Metodi analitici per le acque. **1**, 137–140. *I*.*G*.*E*.*R*. Roma (2003).

[45] Istituto Superiore di Sanità. Reference analytical methods for water intended for human consumption according to the Italian Legislative Decree 31/2001. **4**, 45–47 in *Bonadonna L*, *Ottaviani M* (*ed*.), *Microbiological methods*. *Rapporti ISTISAN07/5* (2007).

[46] Van Kerrebroeck S, Bastos FCC, Harth H, De Vuyst L (2016). A low pH does not determine the community dynamics of spontaneously developed backslopped liquid wheat sourdoughs but does influence their metabolite kinetics. Int. J. Food Microbiol..

[47] Minervini F (2012). Lactic acid bacterium and yeast microbiotas of 19 sourdoughs used for traditional/typical Italian breads: interactions between ingredients and microbial species diversity. Appl. Environ. Microbiol..

[48] Ahmed W, Sawant S, Huygens F, Goonetilleke A, Gardner T (2009). Prevalence and occurrence of zoonotic bacterial pathogens in surface waters determined by quantitative PCR. Water Res..

[49] De Angelis M (2001). Characterization of non-starter lactic acid bacteria from Italian ewe cheeses based on phenotypic, genotypic, and cell wall protein analyses. Appl. Environ. Microbiol..

[50] De Angelis M (2006). Selection of potential probiotic lactobacilli from pig feces to be used as additives in pelleted feeding. Res. Microbiol..

[51] Torriani S, Felis GE, Dellaglio F (2001). Differentiation of *Lactobacillus plantarum*, *L*. *pentosus*, and *L*. *paraplantarum* by *recA* gene sequence analysis and multiplex PCR assay with *recA* gene-derived primers. Appl. Environ. Microbiol..

[52] Naser SM (2005). Application of multilocus sequence analysis (MLSA) for rapid identification of *Enterococcus* species based on *rpoA* and *pheS* genes. Microbiology.

[53] Berthier F, Ehrlich SD (1998). Rapid species identification within two groups of closely related lactobacilli using PCR primers that target the 16S/23S rRNA spacer region. FEMS Microbiol. Lett..

[54] Lattanzi A (2013). The lactic acid bacteria and yeast microbiota of eighteen sourdoughs used for the manufacture of traditional Italian sweet leavened baked goods. Int. J. Food Microbiol..

[55] Muhling M, Woolven-Allen J, Murrell JC (2008). & Joint, I. Improved group-specific PCR primers for denaturing gradient gel electrophoresis analysis of the genetic diversity of complex microbial communities. ISME J..

[56] Zhang J, Kobert K, Flouri T, Stamatakis A (2014). PEAR: a fast and accurate Illumina Paired-End reAd mergeR. Bioinformatics.

[57] Edgar RC, Haas BJ, Clemente JC, Quince C, Knight R (2011). UCHIME improves sensitivity and speed of chimera detection. Bioinformatics.

[58] Edgar RC (2010). Search and clustering orders of magnitude faster than BLAST. Bioinformatics.

[59] Edgar RC (2013). UPARSE: highly accurate OTU sequences from microbial amplicon reads. Nat Methods.

[60] AACC International Method. Guidelines for measurement of volume by rapeseed displacement, 10.1094/AACCIntMethod-10-05.01 (2001).

[61] Crowley P, Grau H, Arendt EK (2000). Influence of additives and mixing time on crumb grain characteristics of wheat bread. Cereal Chem..

[62] Caraux G, Pinloche S (2005). PermutMatrix: a graphical environment to arrange gene expression profiles in optimal linear order. Bioinformatics.

