# Supplementary material for: Tap water is one of the drivers that establish and assembly the lactic acid bacterium biota during sourdough preparation
Source: Sci Rep. 2019 Jan 24;9:570. doi: 10.1038/s41598-018-36786-2 (PMC6345887; doi:10.1038/s41598-018-36786-2)
Supplement: Supplementary file 1 — Supplementary figures and tables [file 41598_2018_36786_MOESM1_ESM.pdf]

SUPPLEMENTARY MATERIAL

Tap water is one of the drivers that establish and assembly the lactic acid bacterium biota during sourdough preparation

Authors: **Fabio Minervini, Francesca Rita Dinardo, Maria De Angelis, Marco Gobbetti**

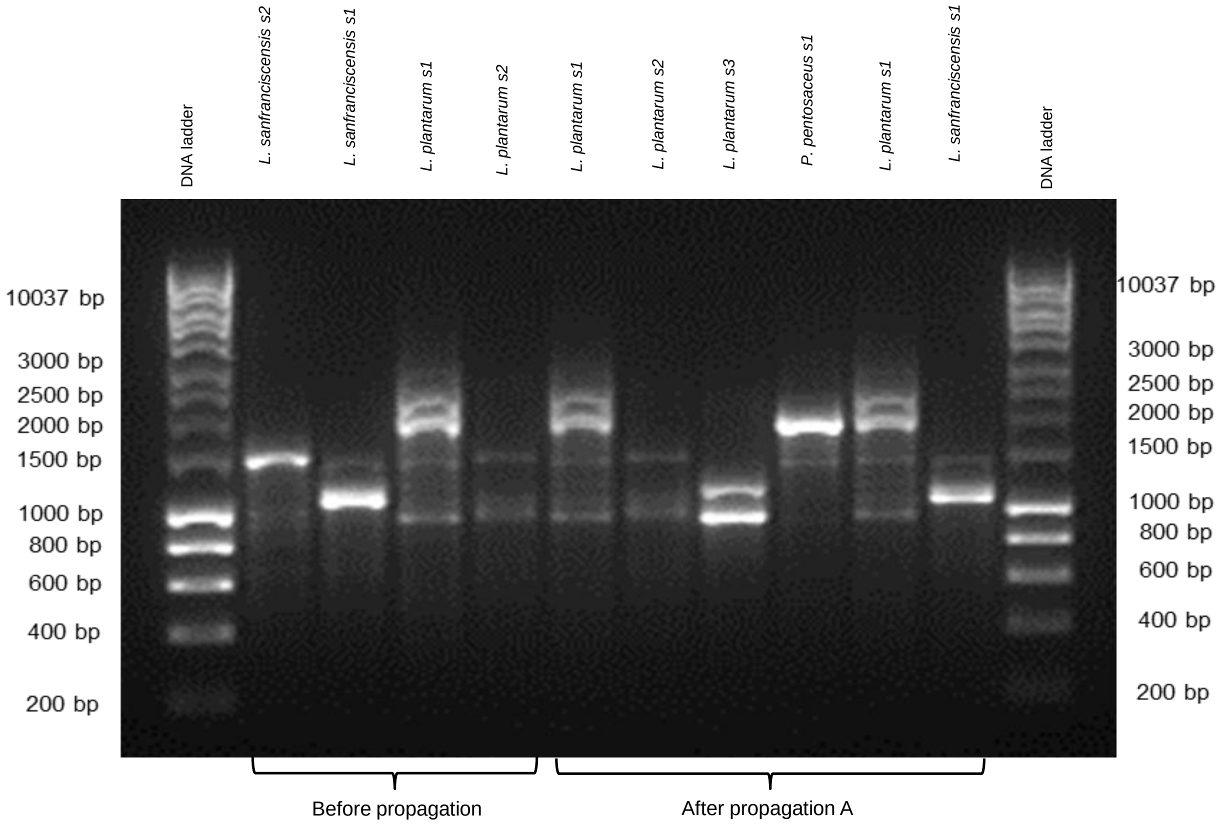

Supplementary figure 1a. Representative randomly amplified polymorphic DNA-PCR (RAPD-PCR) profiles (obtained with M13 primer) of lactic acid bacteria (LAB) isolated from traditional sourdough before propagation and after propagation using water collected in Abruzzo (A). Profiles refer to M13 primer. The HyperLadder™ 1kb (Bioline) (200 – 10037 bp) was used as DNA molecular size standard.

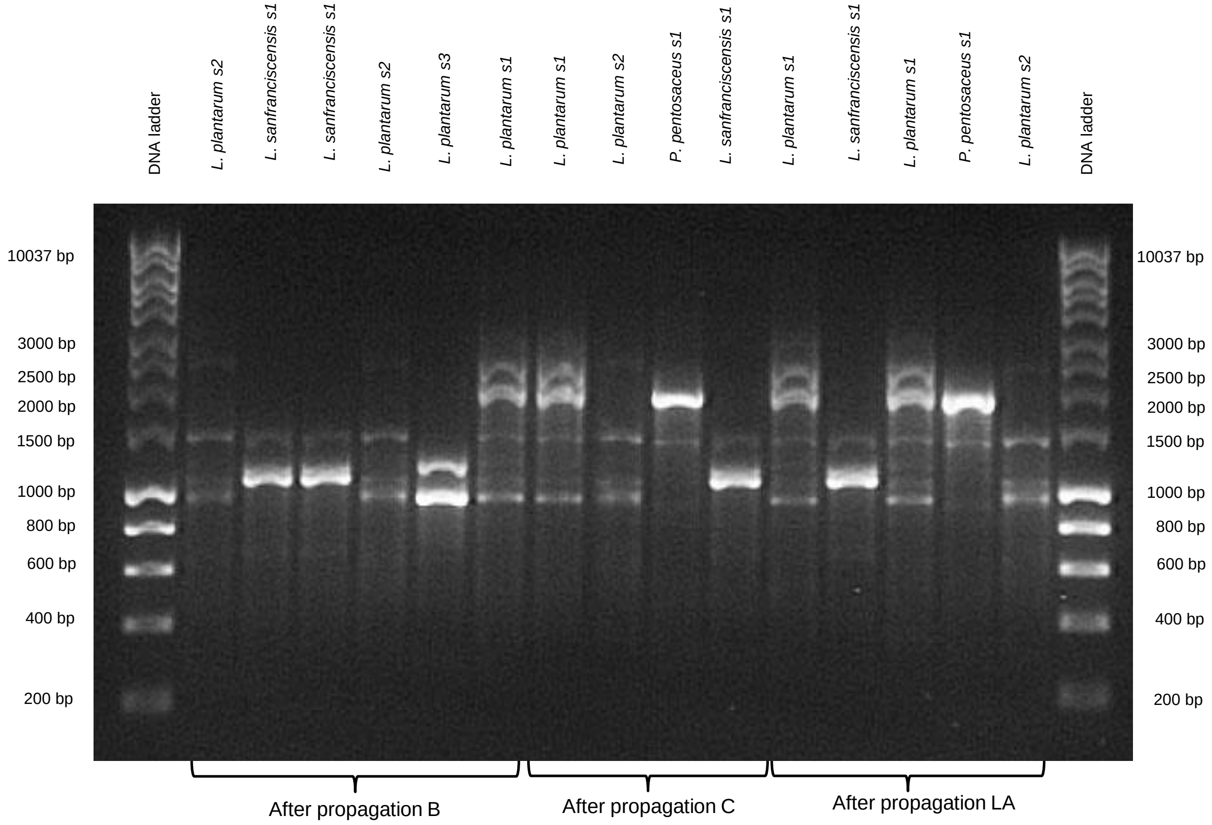

Supplementary figure 1b. Representative randomly amplified polymorphic DNA-PCR (RAPD-PCR) profiles (obtained with M13 primer) of lactic acid bacteria (LAB) isolated from traditional sourdough after propagation using water collected in Basilicata (B), Campania (C) or Lazio (LA). The HyperLadder™ 1kb (Bioline) (200 – 10037 bp) was used as DNA molecular size standard.

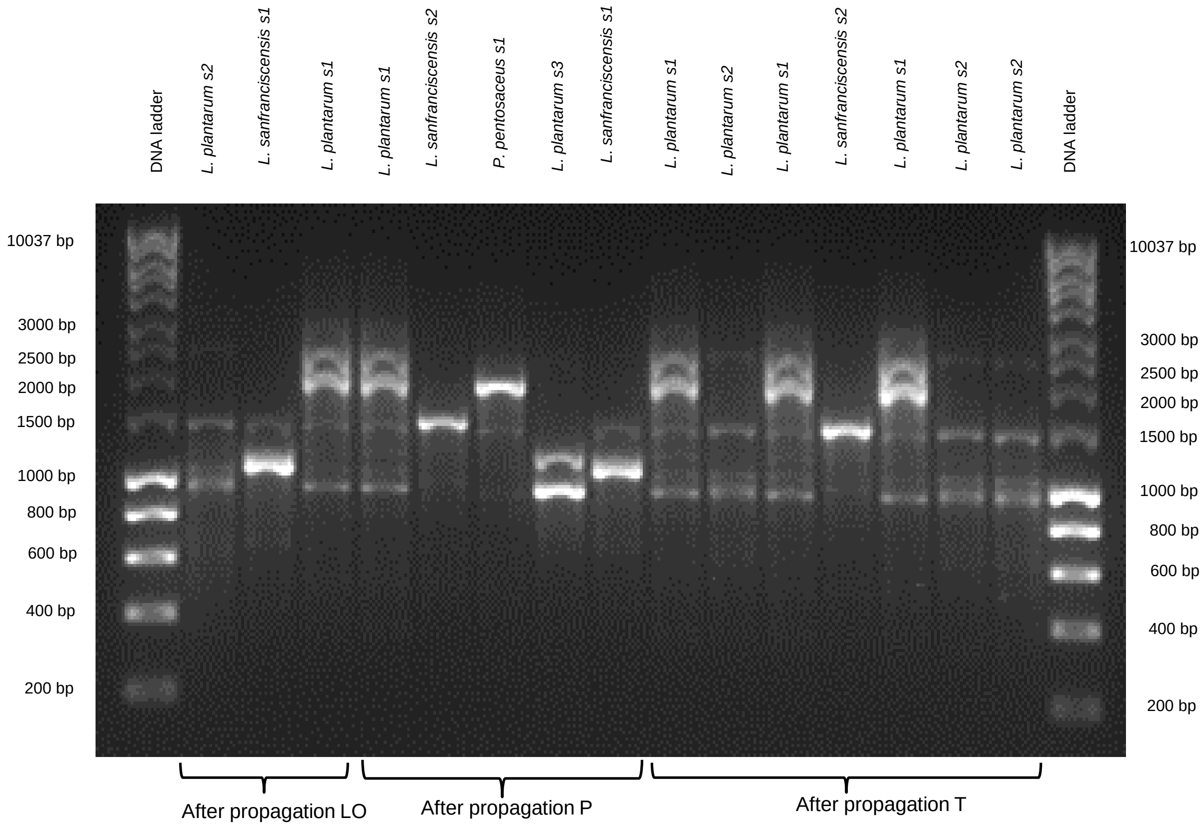

Supplementary figure 1c. Representative randomly amplified polymorphic DNA-PCR (RAPD-PCR) profiles (obtained with M13 primer) of lactic acid bacteria (LAB) isolated from traditional sourdough after propagation using water collected in Lombardia (LO), Puglia (P) or Toscana (T). The HyperLadder™ 1kb (Bioline) (200 – 10037 bp) was used as DNA molecular size standard.

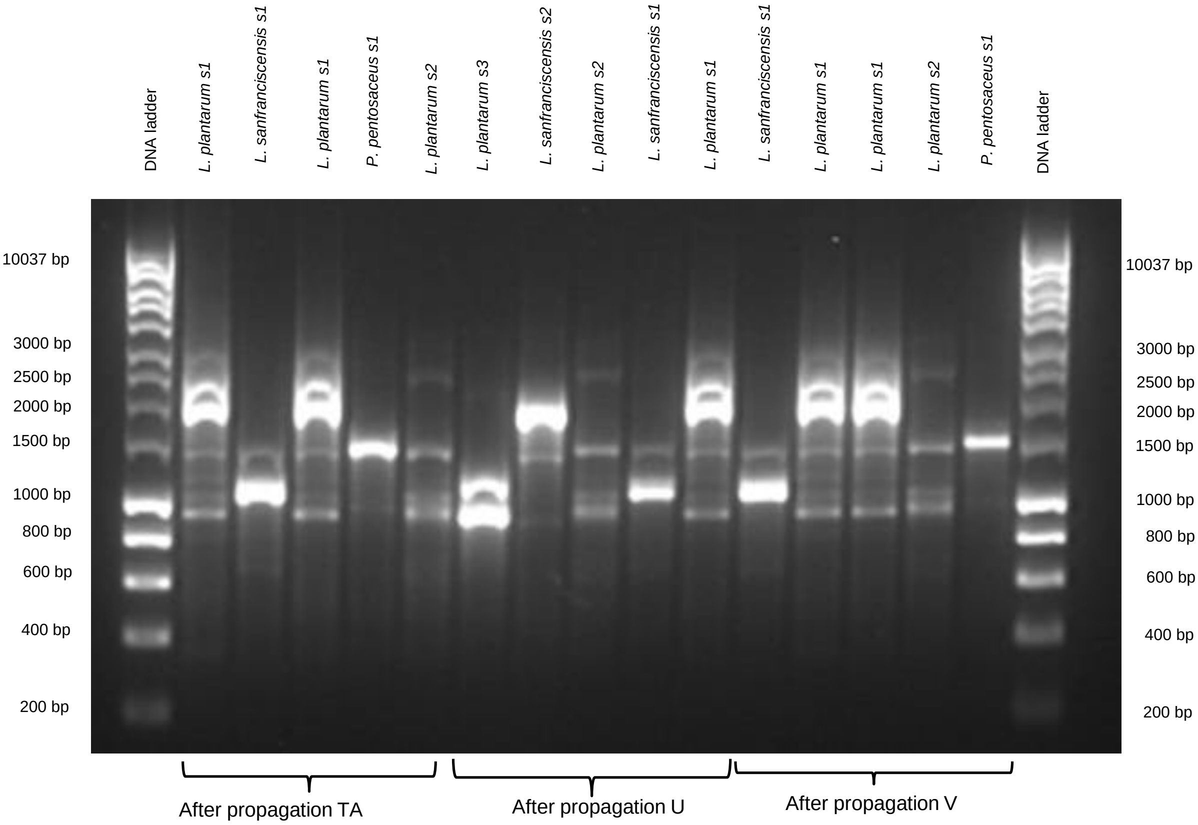

Supplementary figure 1d. Representative randomly amplified polymorphic DNA-PCR (RAPD-PCR) profiles (obtained with M13 primer) of lactic acid bacteria (LAB) isolated from traditional sourdough after propagation using water collected in Trentino-Alto Adige (TA), Umbria (U) or Veneto (V). The HyperLadder™ 1kb (Bioline) (200 – 10037 bp) was used as DNA molecular size standard.

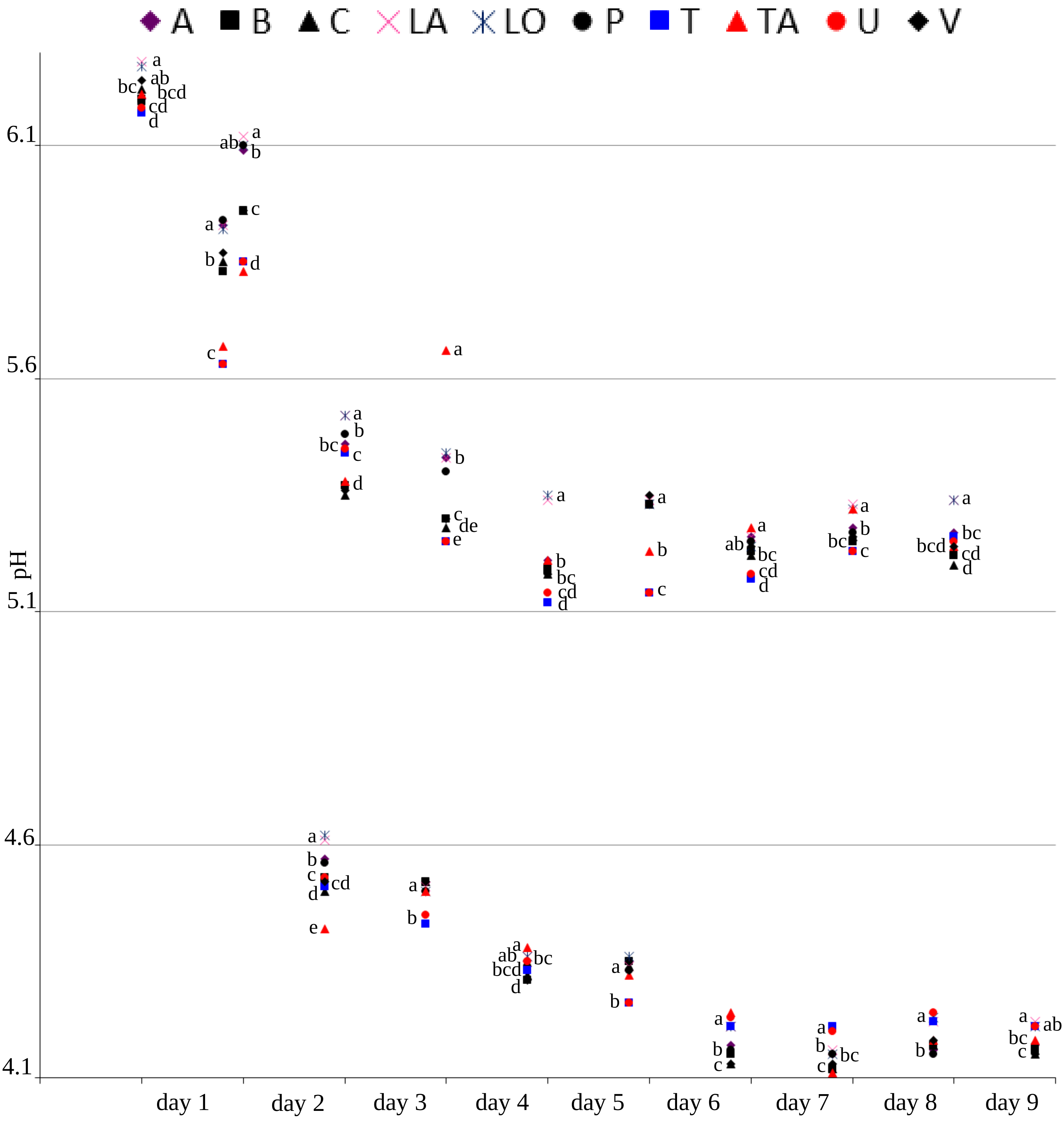

Supplementary figure 2. pH values of doughs and sourdoughs produced using water collected in ten Italian regions. Points at the same day with a common letter were not significantly different at a *P* value of 0.05.

Supplementary table 1. Mean values* (± S.D.) of cell density (log CFU/g) of presumptive lactic acid bacteria (enumerated on mMRS and SDB agar media) in the doughs† produced using water collected in ten Italian regions before fermentation and at day 1 (after 8 h of fermentation at 30 °C); mean values of cell density of presumptive lactic acid bacteria and yeasts (enumerated on SDA) at day 9 (after 6 h of fermentation at 30 °C).

	Before fermentation		Day 1 (8 h of fermentation)		Day 9		
	mMRS	SDB	mMRS	SDB	mMRS	SDB	SDA
A	2.4±0.4a	3.6±0.3a	3.3±0.2c	4.1±0.4c	9.0±0.3c	9.2±0.2ab	5.9±0.2a
B	2.4±0.2a	3.6±0.3a	3.7±0.3ab	4.5±0.4ab	9.3±0.3bc	9.1±0.3ab	5.8±0.1a
C	2.5±0.1a	3.5±0.4a	3.7±0.1ab	4.5±0.3ab	9.5±0.3ab	9.2±0.2ab	6.0±0.2a
LA	2.6±0.2a	3.5±0.2a	3.5±0.2c	4.6±0.3ab	9.5±0.4ab	9.1±0.3ab	6.0±0.2a
LO	2.4±0.3a	3.5±0.2a	3.5±0.2ab	4.6±0.4ab	9.3±0.3b	9.0±0.3b	5.9±0.1a
P	2.5±0.4a	3.6±0.4a	3.3±0.1c	4.1±0.4c	8.9±0.3c	9.1±0.2ab	5.7±0.2a
T	2.5±0.3a	3.4±0.2a	4.0±0.4a	4.8±0.3a	9.1±0.2bc	9.1±0.2ab	5.9±0.3a
TA	2.4±0.2a	3.6±0.3a	3.6±0.2ab	4.9±0.2a	9.8±0.4a	9.6±0.3a	5.8±0.2a
U	2.6±0.4a	3.4±0.4a	3.6±0.3ab	4.9±0.3a	9.1±0.3bc	9.1±0.3ab	6.0±0.3a
V	2.5±0.2a	3.4±0.1a	3.7±0.2ab	4.5±0.2c	9.3±0.2bc	9.1±0.3ab	5.7±0.2a

* Mean (from three analytical replicates) values in the same column with a common letter are not significantly different at a *P* value of 0.05.

† The name of each dough derived from the tap water used during the preparation of sourdough..

Supplementary table 2. Species and strains of lactic acid bacteria isolated from the doughs† produced using water collected in ten Italian regions, at day 1 (after 8 h of fermentation at 30 °C). The dot indicates the presence of strains.

	A	B	C	LA	LO	P	T	TA	U	V
Lactococcus lactis s1	●	●	●	●	●	●	●	●	●	●
Leuconostoc citreum s1		●								
Ln. citreum s2	●	●	●	●	●	●	●	●	●	●
Ln. citreum s3	●	●	●	●	●	●	●	●	●	●
Leuconostoc pseudomesenteroides s1		●					●	●		
Weissella confusa s1	●	●	●	●	●	●	●	●	●	●
W. confusa s2	●	●	●	●	●		●	●	●	●
W. confusa s3						●				
W. confusa s4	●		●					●		
W. confusa s5					●			●		●
W. confusa s6	●			●		●	●			
W. confusa s7				●	●					

† The name of each dough derived from the tap water used during the preparation of sourdough.

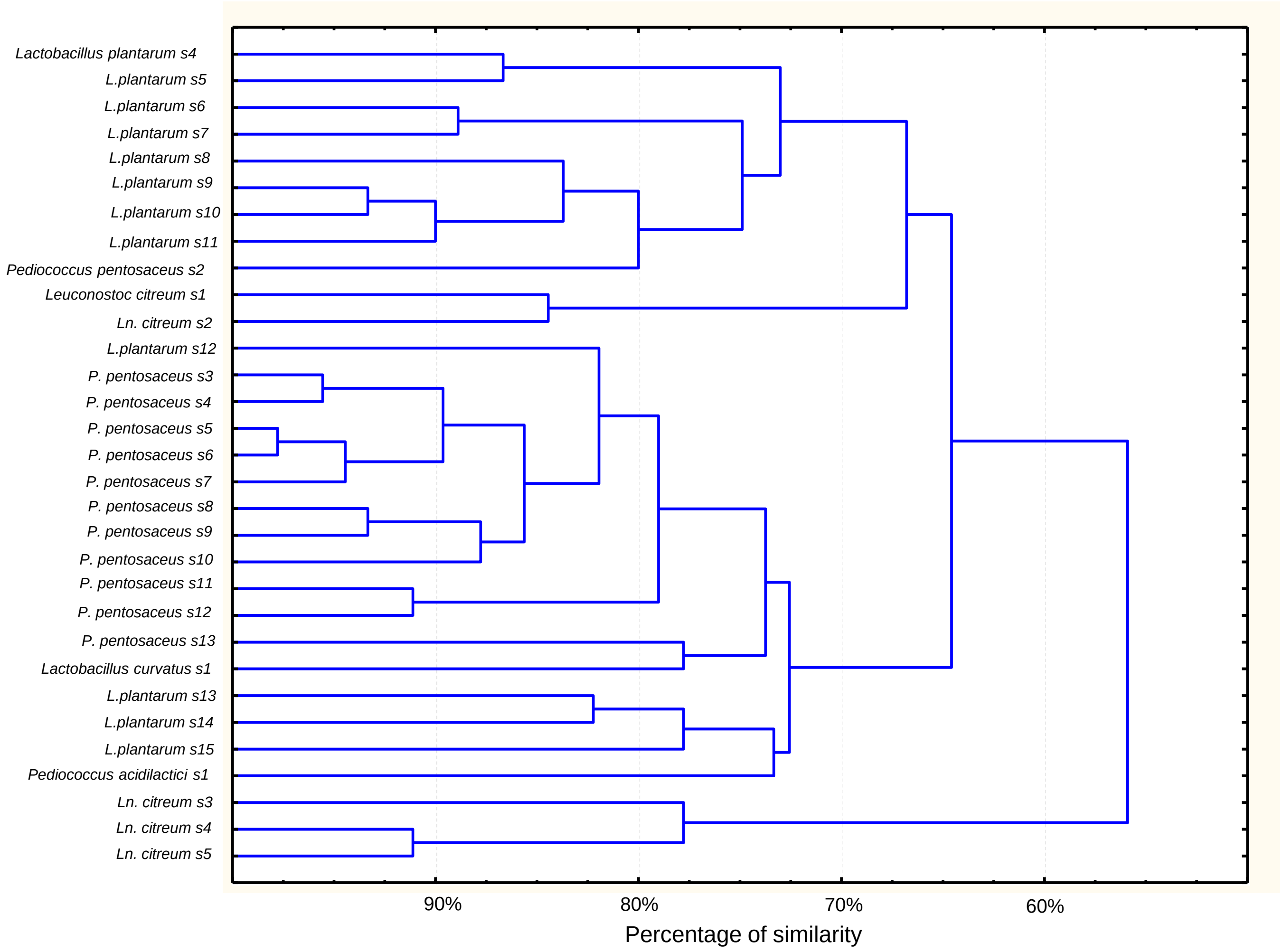

Supplementary figure 3. Dendrogram of combined (primers P4, P7 and M13) RAPD profiles of lactic acid bacterium strains isolated from the sourdoughs prepared at laboratory using water collected in ten Italian regions.

Supplementary table 3. Physical characteristics of breads made using sourdoughs newly prepared using water collected in ten Italian regions.

Sourdough bread[a]	Hardness (N)	Elasticity (%)	Gumminess (N)	Chewiness (N)	Cohesiveness	Specific volume (cm^3/g)	Black pixel area[b] (%)
A	19.64±1.2d	60±5a	30.27±4.78b	29.71±6.50a	0.23±0.02a	2.50±0.33a	53±1.9a
B	17.30±0.8e	54±7a	30.06±6.23b	24.08±3.17b	0.19±0.02a	2.61±0.18a	50±1.8a
C	30.08±2.2a	57±6a	40.63±6.10a	28.95±2.52a	0.22±0.03a	2.41±0.30a	51±2.1a
LA	26.20±1.0b	56±6a	42.50±5.16a	31.82±4.01a	0.25±0.04a	2.45±0.16a	50±2.4a
LO	15.54±0.5f	60±4a	26.14±9.44b	25.15±3.50b	0.20±0.03a	2.43±0.19a	52±2.0a
P	15.73±0.8f	59±5a	26.03±8.69b	12.75±2.09d	0.25±0.02a	2.54±0.25a	55±2.5a
T	17.75±0.9e	57±5a	31.23±5.70b	25.84±4.30b	0.27±0.04a	2.49±0.21a	52±2.0a
TA	17.81±0.6e	54±4a	29.55±4.64b	17.41±4.10c	0.23±0.04a	2.44±0.30a	51±2.3a
U	24.85±0.9c	58±5a	39.20±5.56a	30.15±3.60a	0.26±0.04a	2.46±0.19a	54±2.1a
V	22.16±1.0d	60±6a	31.86±4.50b	24.70±3.20b	0.26±0.03a	2.47±0.25a	51±2.6a

[a] The name of each sourdough bread derived from the tap water used during the preparation of sourdough.

[b] Black pixel area was the percentage of the image area filled up with black pixel (gas cells).

Mean values (±S.D.) in the same column with a common letter were not significantly different at a *P* value of 0.05.
